# Supplementary material for: Application of Iodine as a Catalyst in Aerobic Oxidations: A Sustainable Approach for Thiol Oxidations
Source: Molecules. 2023 Sep 24;28(19):6789. doi: 10.3390/molecules28196789 (PMC10574728; doi:10.3390/molecules28196789)
Supplement: Supplementary file 1 [file molecules-28-06789-s001.zip › molecules-2622088-supplementary.pdf]

Supplementary Materials

Application of Iodine as a Catalyst in Aerobic Oxidations: A Sustainable Approach for Thiol Oxidations

Lijun Wang [1], Lingxia Chen [2], Zixuan Qin [2], Ke Ni[2], Xiao Li[1], Zhiyuan Yu [1], Zichen Kuang[1], Xinshu Qin [2], Hongxia Duan [1,*] and Jie An [2,*]

[1] Department of Chemistry and Innovation Center of Pesticide Research, College of Science, China Agricultural University, Beijing 100193, China; lijunwang@cau.edu.cn (L.J.W.); 1824204541@qq.com (X.L.); 1459569986@qq.com (Z.Y.Y); kuangzichen@126.com (Z.C.K.); hxduan@cau.edu.cn (H.X.D.)

[2] Department of Nutrition and Health, China Agricultural University, Beijing, 100083, China; lxchen915@163.com (L.X.C.); shxzq1@nottingham.edu.cn (Z.X.Q.); qinxs98@163.com (X.S.Q.); ke.ni@pepperdine.edu (K.N.);

* Correspondence: hxduan@cau.edu.cn; jie_an@cau.edu.cn

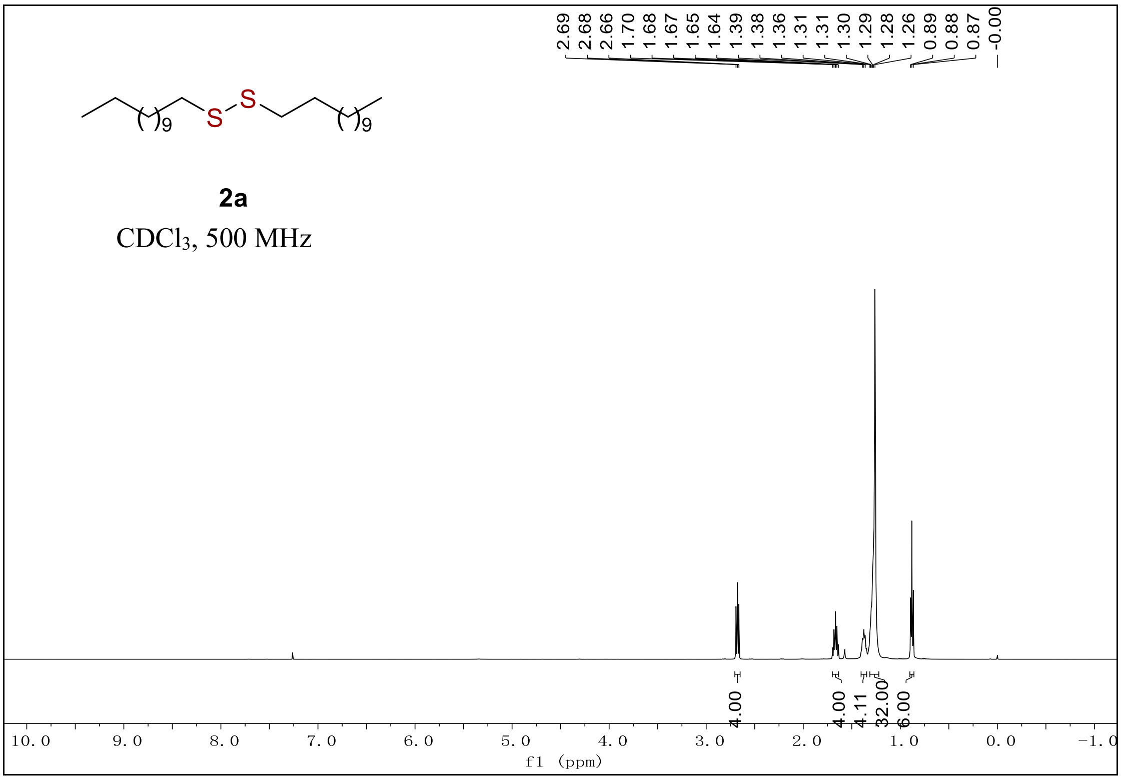

2.69
2.68
2.66
1.70
1.68
1.67
1.65
1.64
1.39
1.38
1.36
1.31
1.31
1.30
1.29
1.28
1.26
0.89
0.88
0.87
-0.00
S
S
9
9
2a
CDCl3, 500 MHz
4.00
4.00
4.11
32.00
6.00
10.0
9.0
8.0
7.0
6.0
5.0
4.0
3.0
2.0
1.0
0.0
-1.0
f1 (ppm)

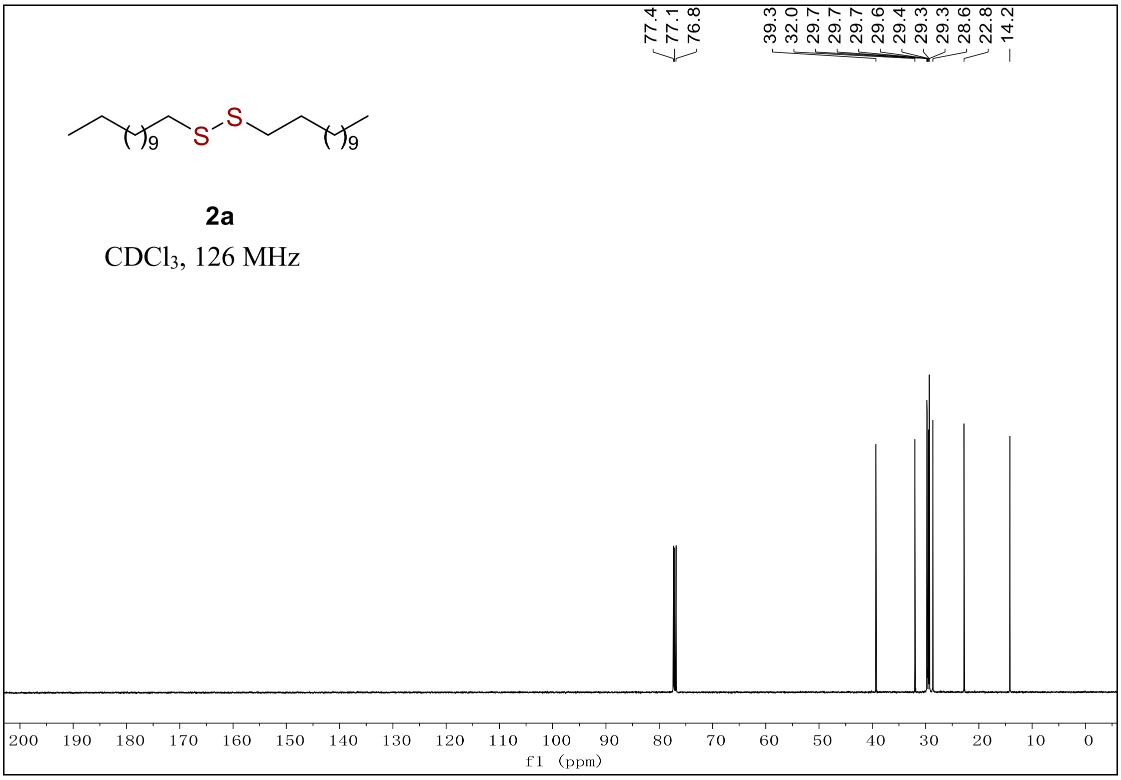

77.4
77.1
76.8
39.3
32.0
29.7
29.7
29.7
29.6
29.4
29.3
29.3
28.6
22.8
14.2
S
S
9
9
2a
CDCl3, 126 MHz
200
190
180
170
160
150
140
130
120
110
100
90
80
70
60
50
40
30
20
10
0
f1 (ppm)

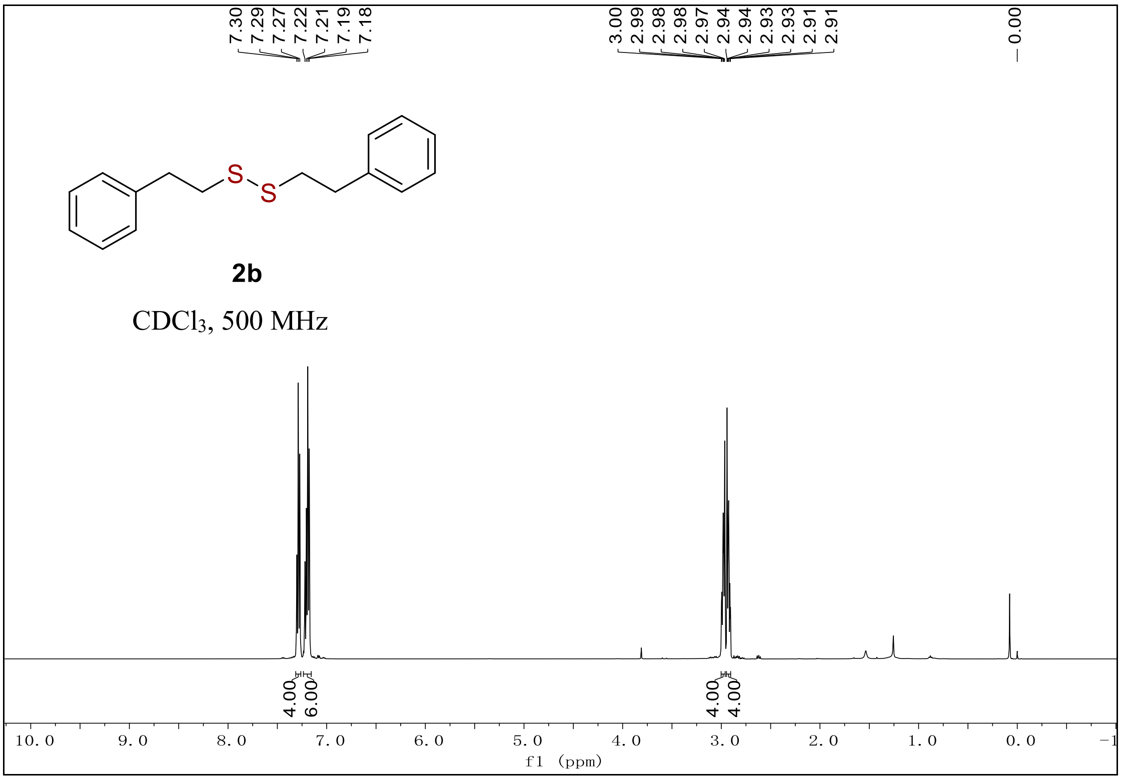
7.30 7.29 7.27 7.22 7.21 7.19 7.18
3.00 2.99 2.98 2.98 2.97 2.94 2.94 2.93 2.93 2.91 2.91
0.00
S
S
2b
CDCl3, 500 MHz
4.00 6.00
4.00 4.00
10.0 9.0 8.0 7.0 6.0 5.0 4.0 3.0 2.0 1.0 0.0
f1 (ppm)

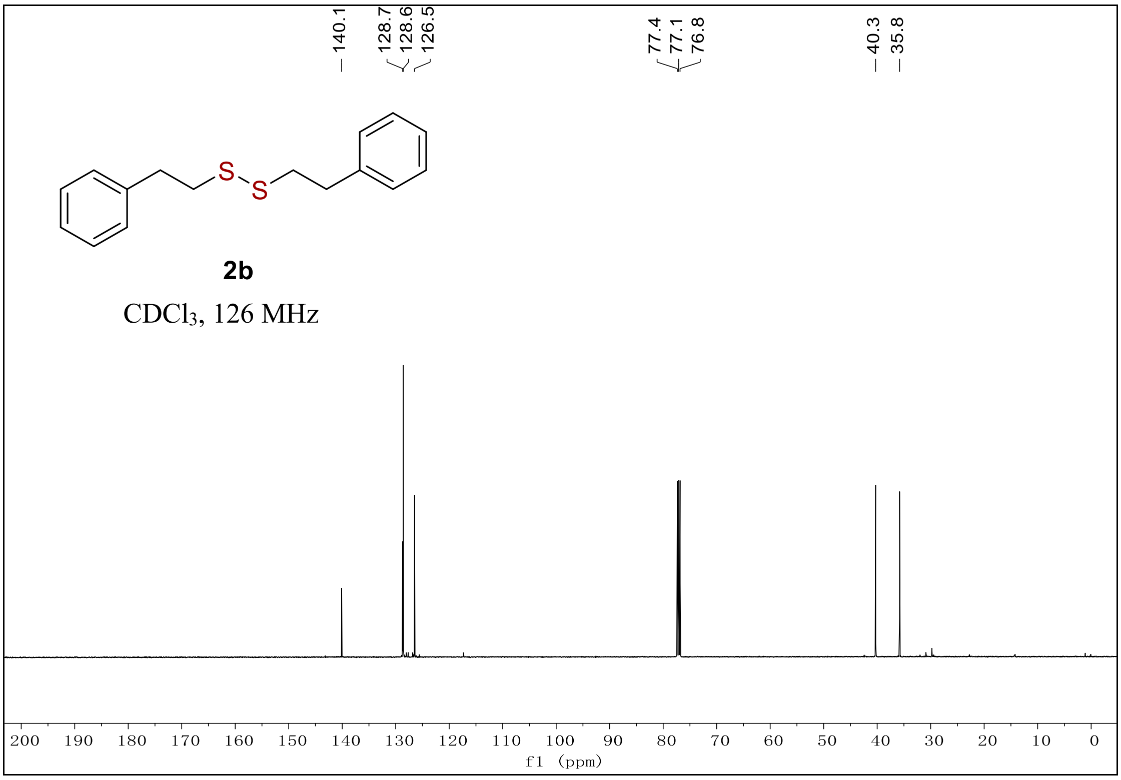
140.1
128.7 128.6 126.5
77.4 77.1 76.8
40.3 35.8
S
S
2b
CDCl3, 126 MHz
200 190 180 170 160 150 140 130 120 110 100 90 80 70 60 50 40 30 20 10 0
f1 (ppm)

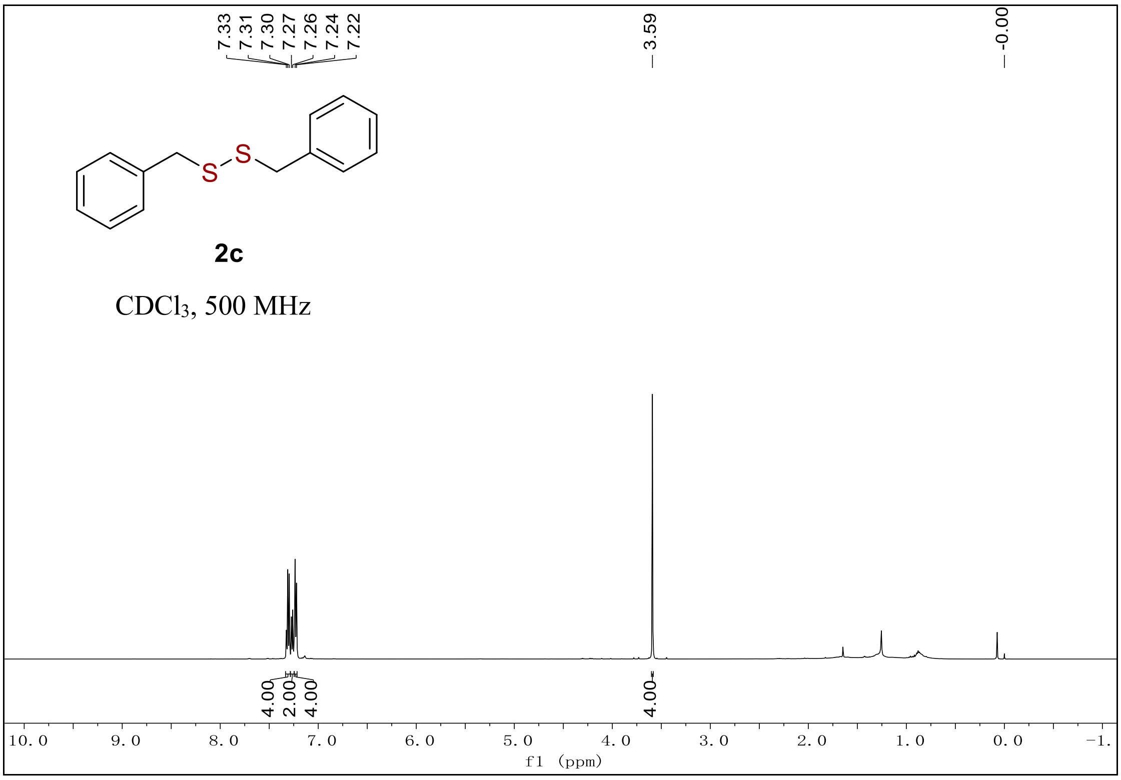
7.33
7.31
7.30
7.27
7.26
7.24
7.22
3.59
-0.00
2c
CDCl3, 500 MHz
4.00
2.00
4.00
4.00
10.0 9.0 8.0 7.0 6.0 5.0 4.0 3.0 2.0 1.0 0.0 -1.
f1 (ppm)

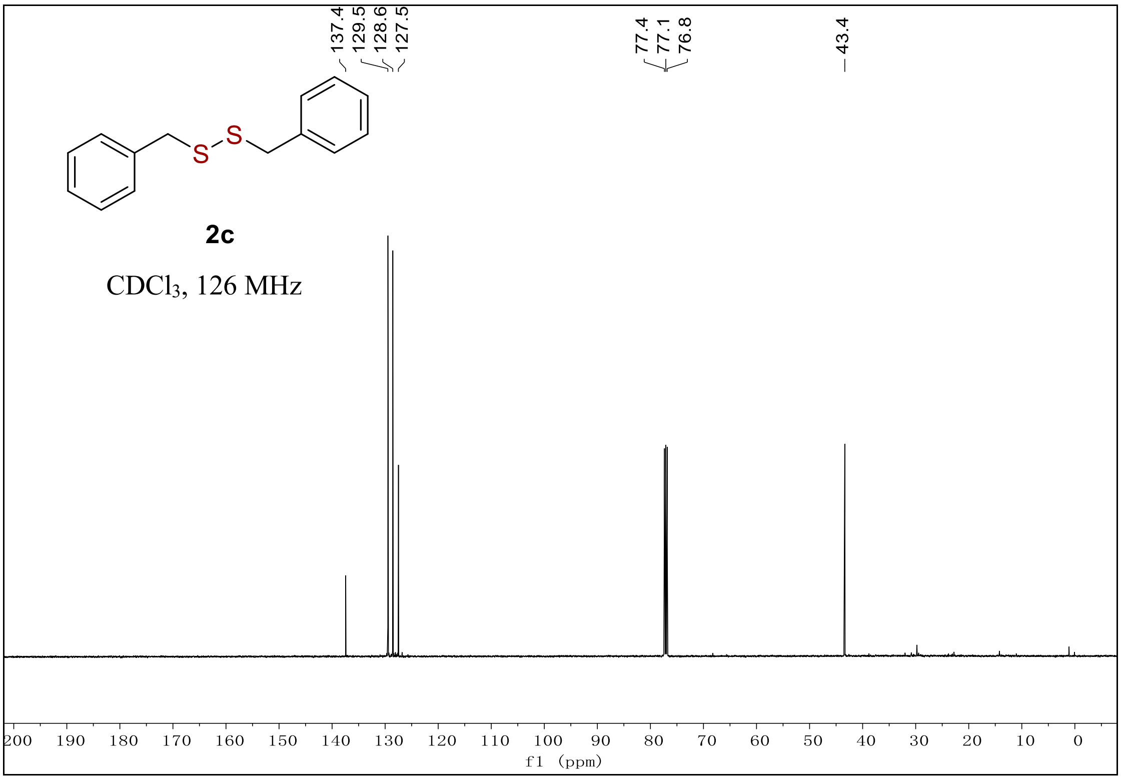
137.4
129.5
128.6
127.5
77.4
77.1
76.8
43.4
2c
CDCl3, 126 MHz
200 190 180 170 160 150 140 130 120 110 100 90 80 70 60 50 40 30 20 10 0
f1 (ppm)

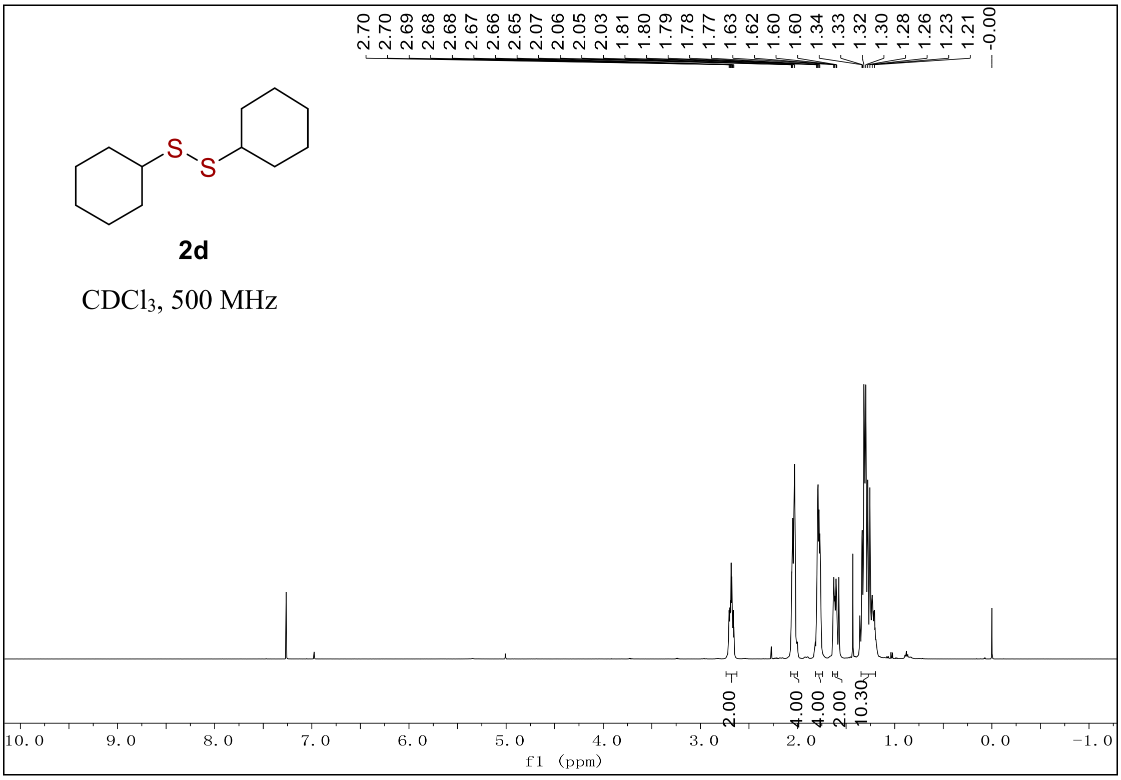
2.70
2.70
2.69
2.68
2.68
2.67
2.66
2.65
2.07
2.06
2.05
2.03
1.81
1.80
1.79
1.78
1.77
1.63
1.62
1.60
1.60
1.34
1.33
1.32
1.30
1.28
1.26
1.23
1.21
-0.00
S
S
2d
CDCl3, 500 MHz
2.00
4.00
4.00
2.00
10.30
10.0
9.0
8.0
7.0
6.0
5.0
4.0
3.0
2.0
1.0
0.0
-1.0
f1 (ppm)

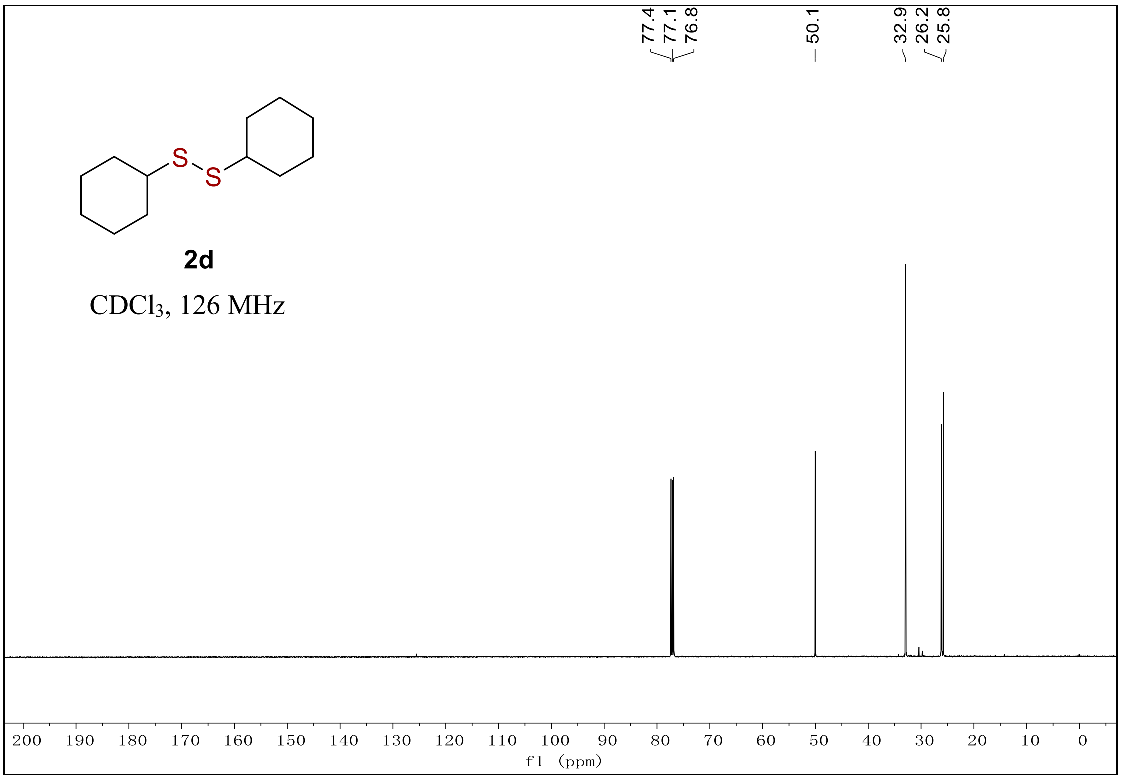
77.4
77.1
76.8
50.1
32.9
26.2
25.8
S
S
2d
CDCl3, 126 MHz
200
190
180
170
160
150
140
130
120
110
100
90
80
70
60
50
40
30
20
10
0
f1 (ppm)

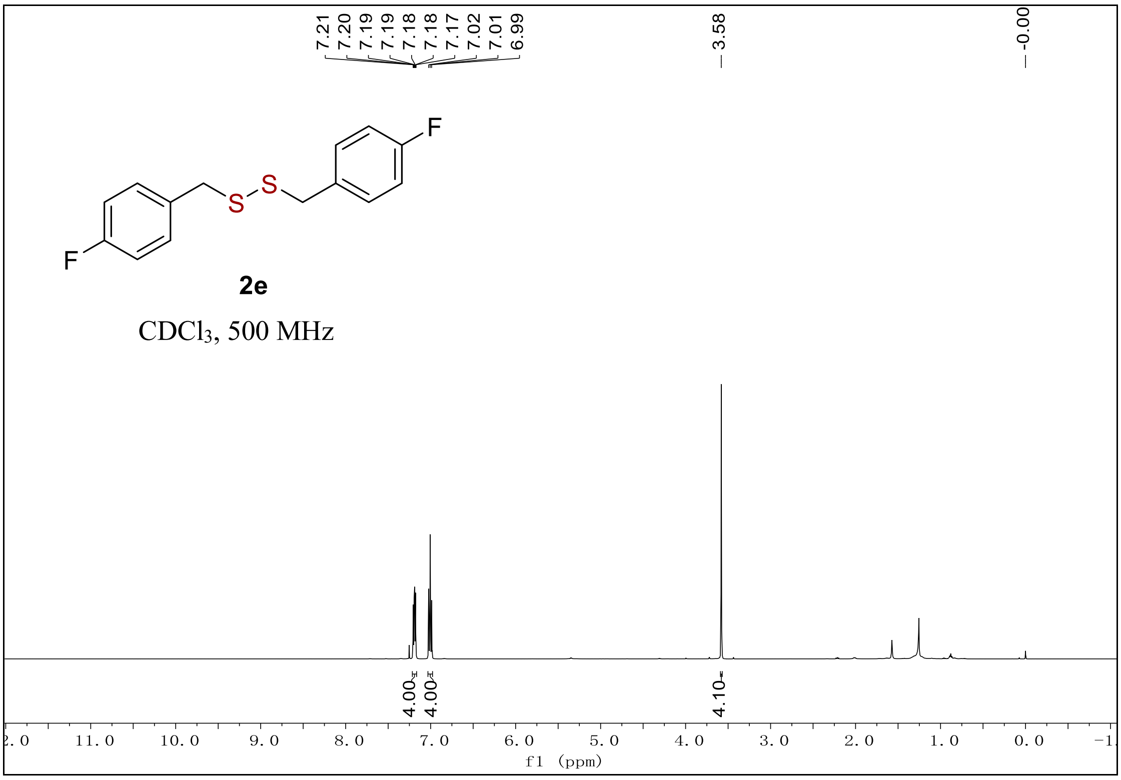
7.21
7.20
7.19
7.19
7.18
7.18
7.17
7.02
7.01
6.99
3.58
-0.00
F
S
S
F
2e
CDCl3, 500 MHz
4.00
4.00
4.10
11.0
10.0
9.0
8.0
7.0
6.0
5.0
4.0
3.0
2.0
1.0
0.0
f1 (ppm)

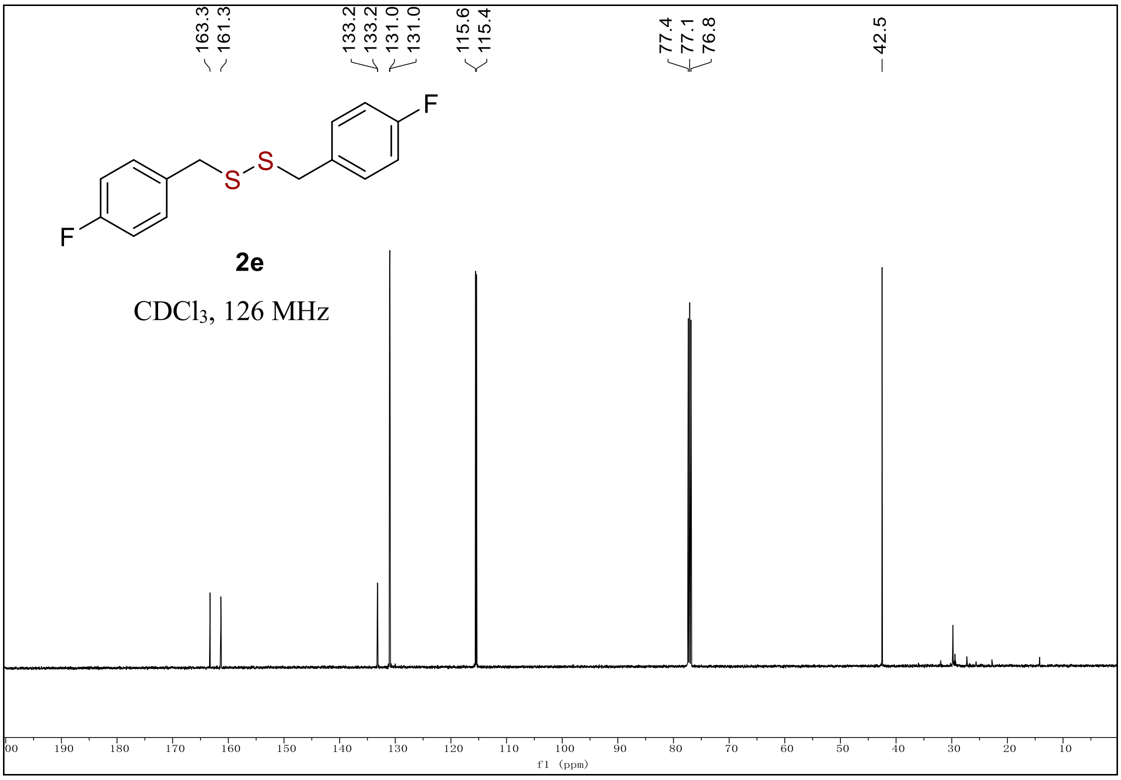
163.3
161.3
133.2
133.2
131.0
131.0
115.6
115.4
77.4
77.1
76.8
42.5
F
S
S
F
2e
CDCl3, 126 MHz
190
180
170
160
150
140
130
120
110
100
90
80
70
60
50
40
30
20
10
f1 (ppm)

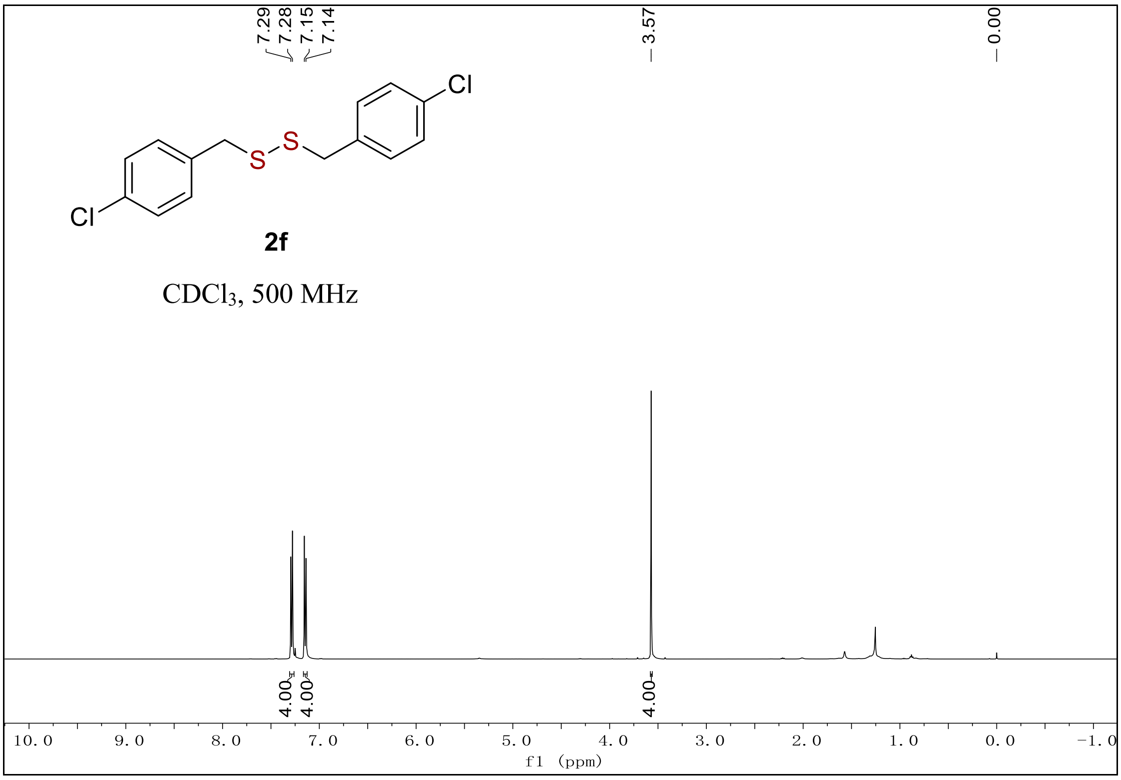
7.29
7.28
7.15
7.14
3.57
0.00
Cl
S
S
Cl
2f
CDCl3, 500 MHz
4.00
4.00
4.00
10.0
9.0
8.0
7.0
6.0
5.0
4.0
3.0
2.0
1.0
0.0
-1.0
f1 (ppm)

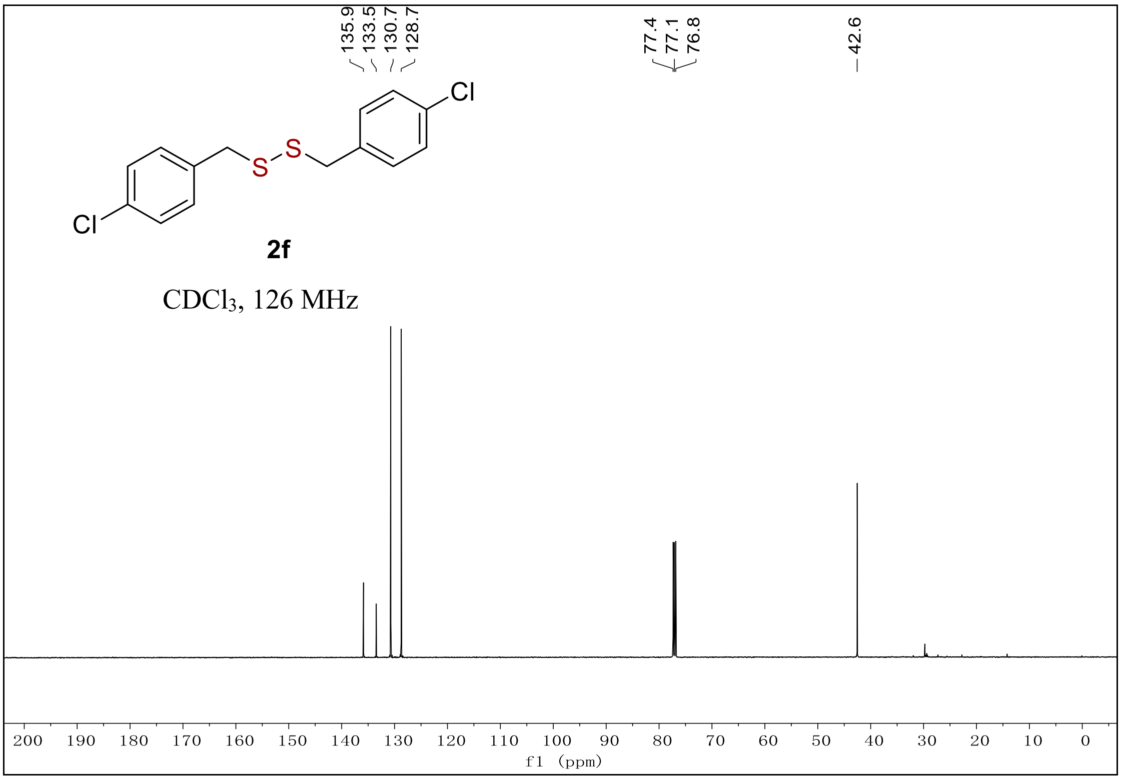
135.9
133.5
130.7
128.7
77.4
77.1
76.8
42.6
Cl
S
S
Cl
2f
CDCl3, 126 MHz
200
190
180
170
160
150
140
130
120
110
100
90
80
70
60
50
40
30
20
10
0
f1 (ppm)

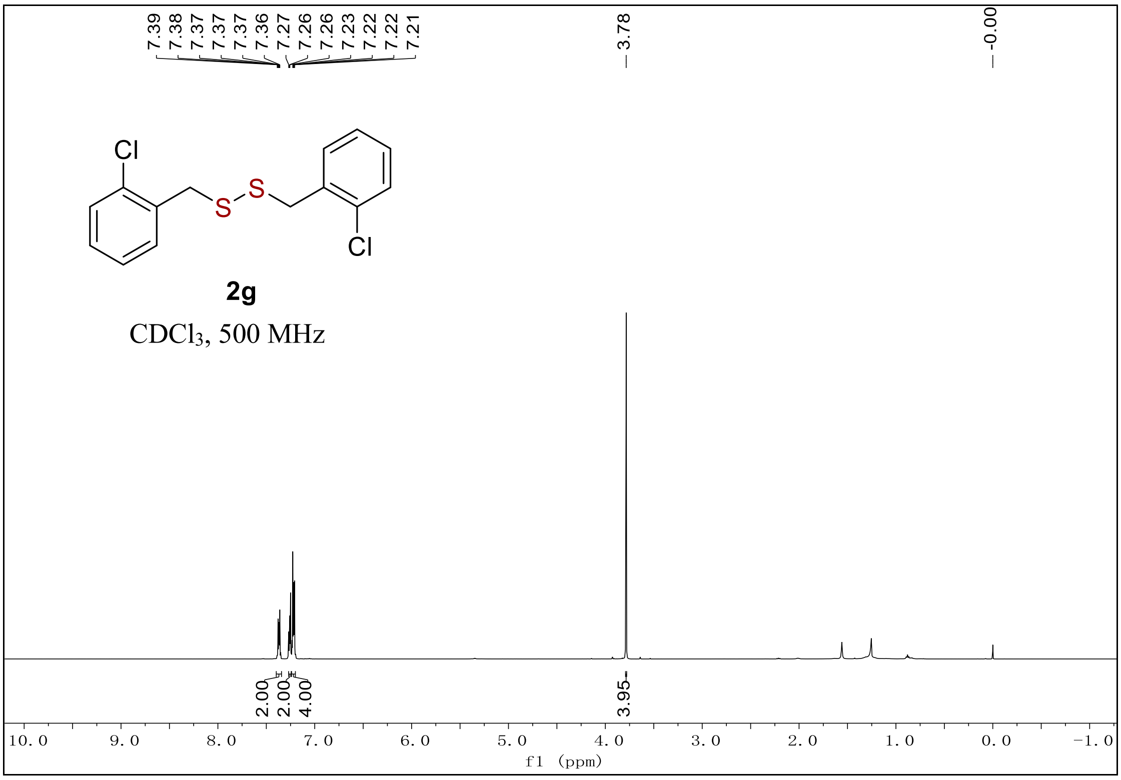

7.39 7.38 7.37 7.37 7.37 7.36 7.27 7.26 7.26 7.23 7.22 7.22 7.21
3.78
-0.00
Cl
S
S
Cl
2g
CDCl3, 500 MHz
2.00 2.00 4.00
3.95
10.0 9.0 8.0 7.0 6.0 5.0 4.0 3.0 2.0 1.0 0.0 -1.0
f1 (ppm)

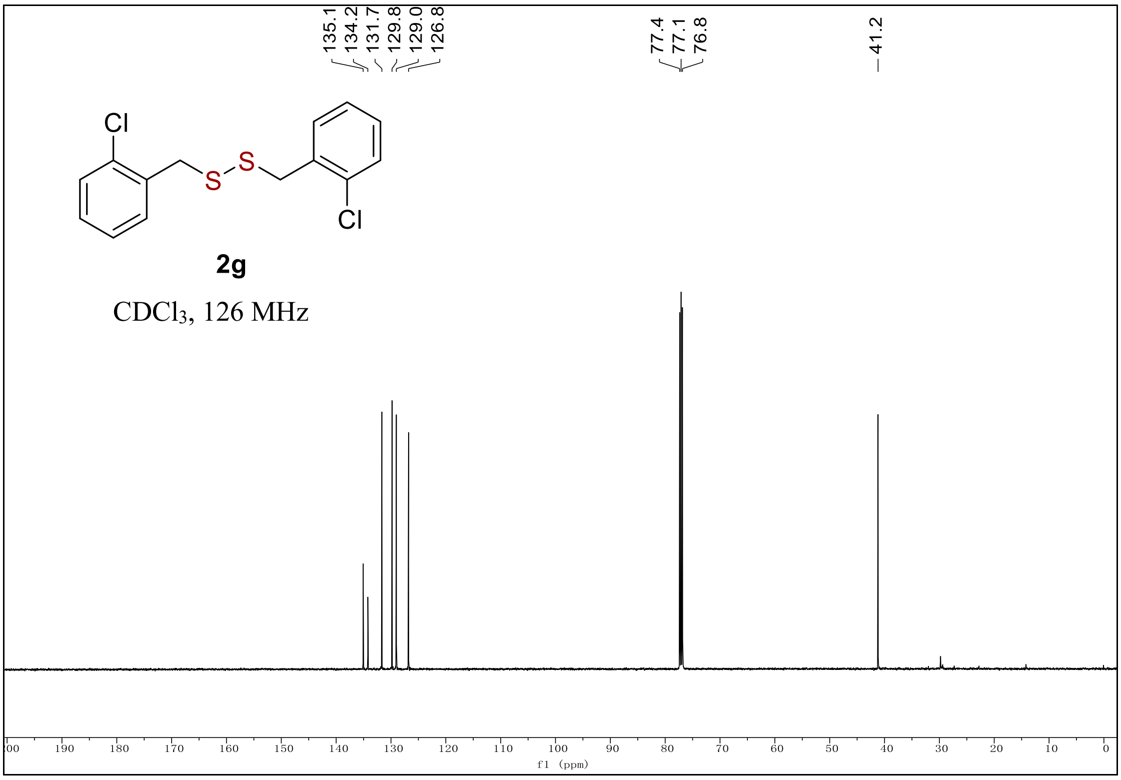

135.1 134.2 131.7 129.8 129.0 126.8
77.4 77.1 76.8
41.2
Cl
S
S
Cl
2g
CDCl3, 126 MHz
190 180 170 160 150 140 130 120 110 100 90 80 70 60 50 40 30 20 10 0
f1 (ppm)

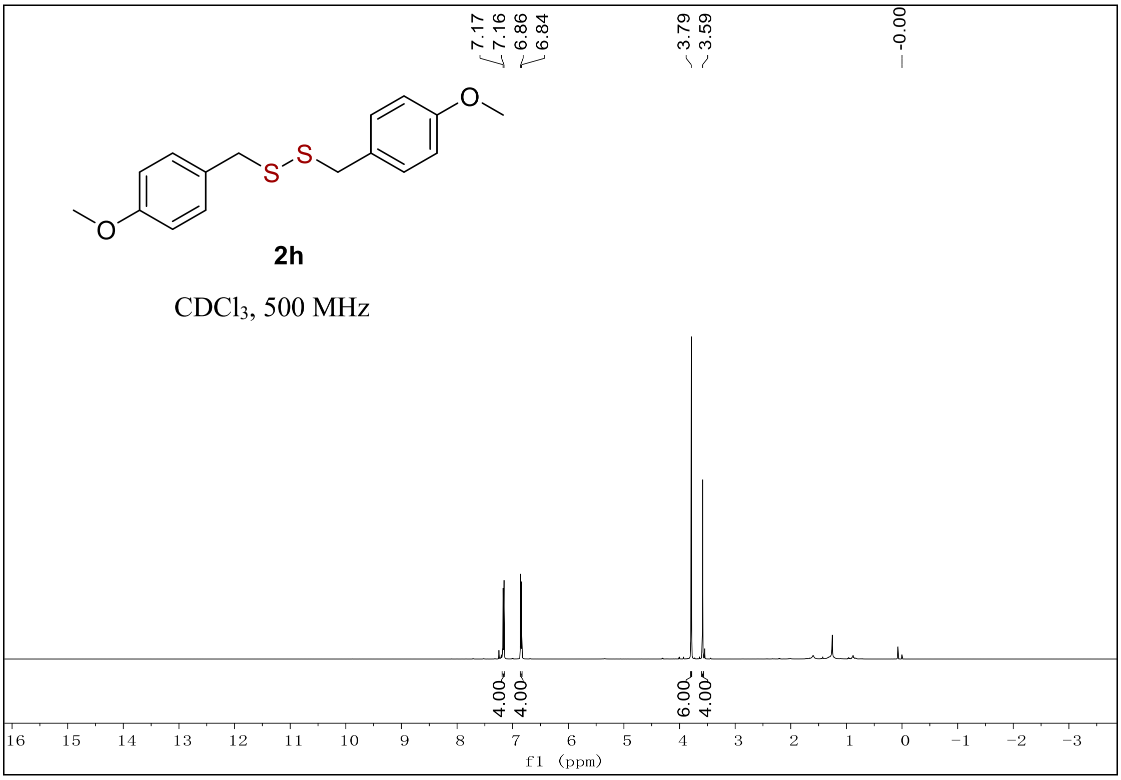
7.17
7.16
6.86
6.84
3.79
3.59
-0.00
O
S
S
O
2h
CDCl3, 500 MHz
4.00
4.00
6.00
4.00
16 15 14 13 12 11 10 9 8 7 6 5 4 3 2 1 0 −1 −2 −3
f1 (ppm)

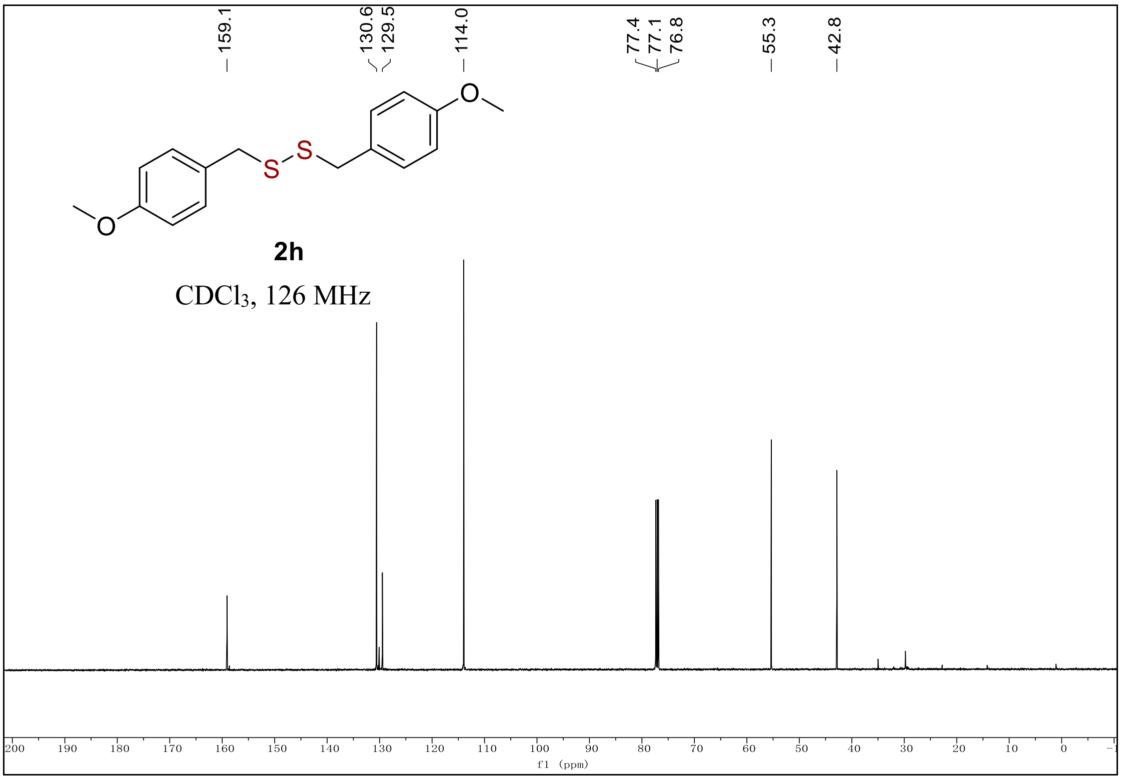
159.1
130.6
129.5
114.0
77.4
77.1
76.8
55.3
42.8
O
S
S
O
2h
CDCl3, 126 MHz
200 190 180 170 160 150 140 130 120 110 100 90 80 70 60 50 40 30 20 10 0
f1 (ppm)

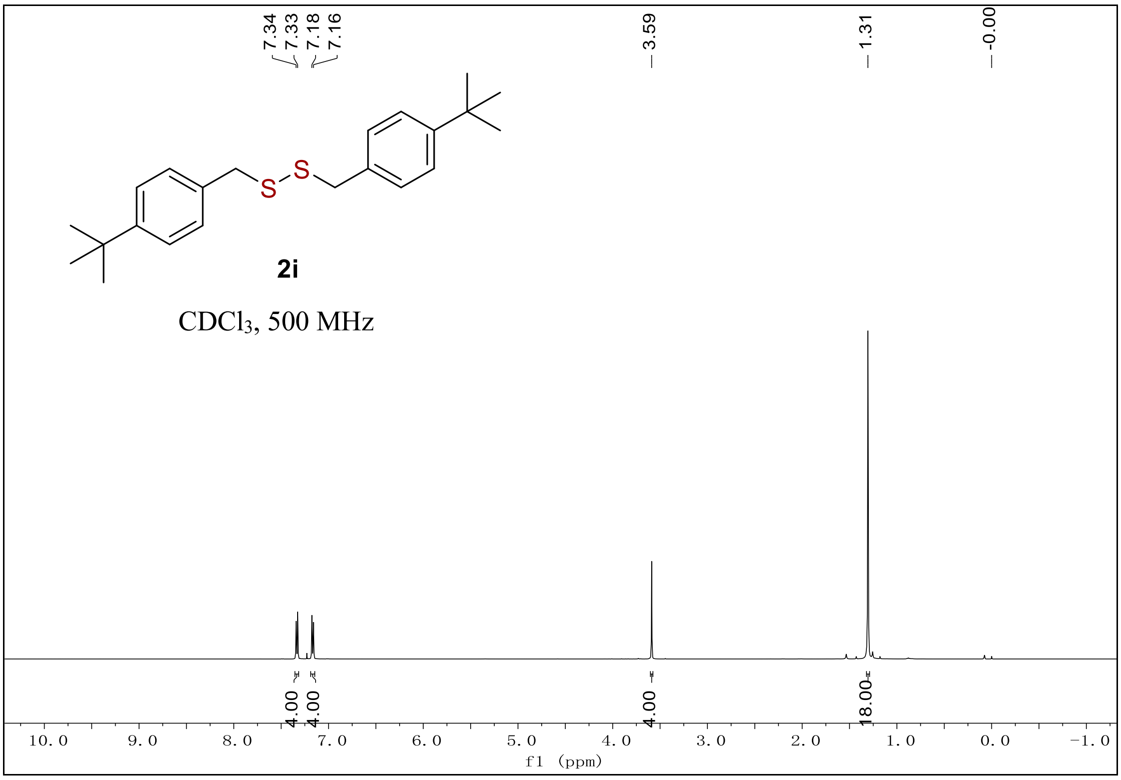
7.34
7.33
7.18
7.16
3.59
1.31
-0.00
2i
CDCl3, 500 MHz
4.00
4.00
4.00
18.00
10.0 9.0 8.0 7.0 6.0 5.0 4.0 3.0 2.0 1.0 0.0 -1.0
f1 (ppm)

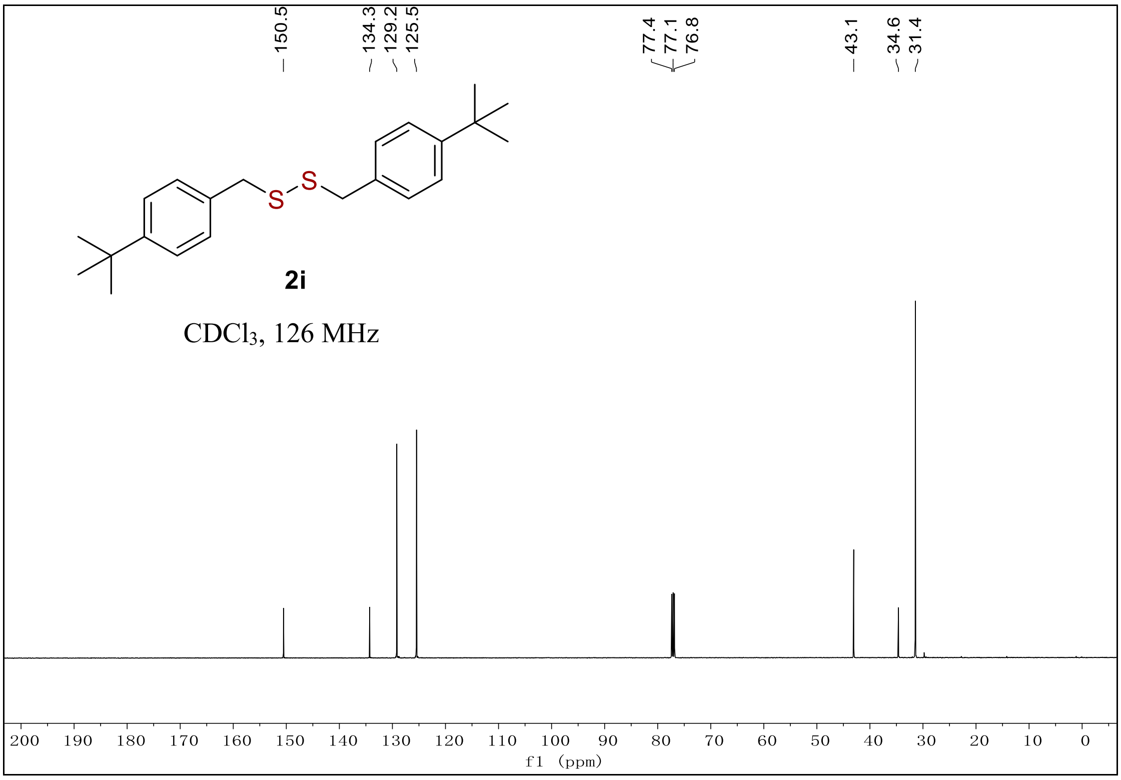
150.5
134.3
129.2
125.5
77.4
77.1
76.8
43.1
34.6
31.4
2i
CDCl3, 126 MHz
200 190 180 170 160 150 140 130 120 110 100 90 80 70 60 50 40 30 20 10 0
f1 (ppm)

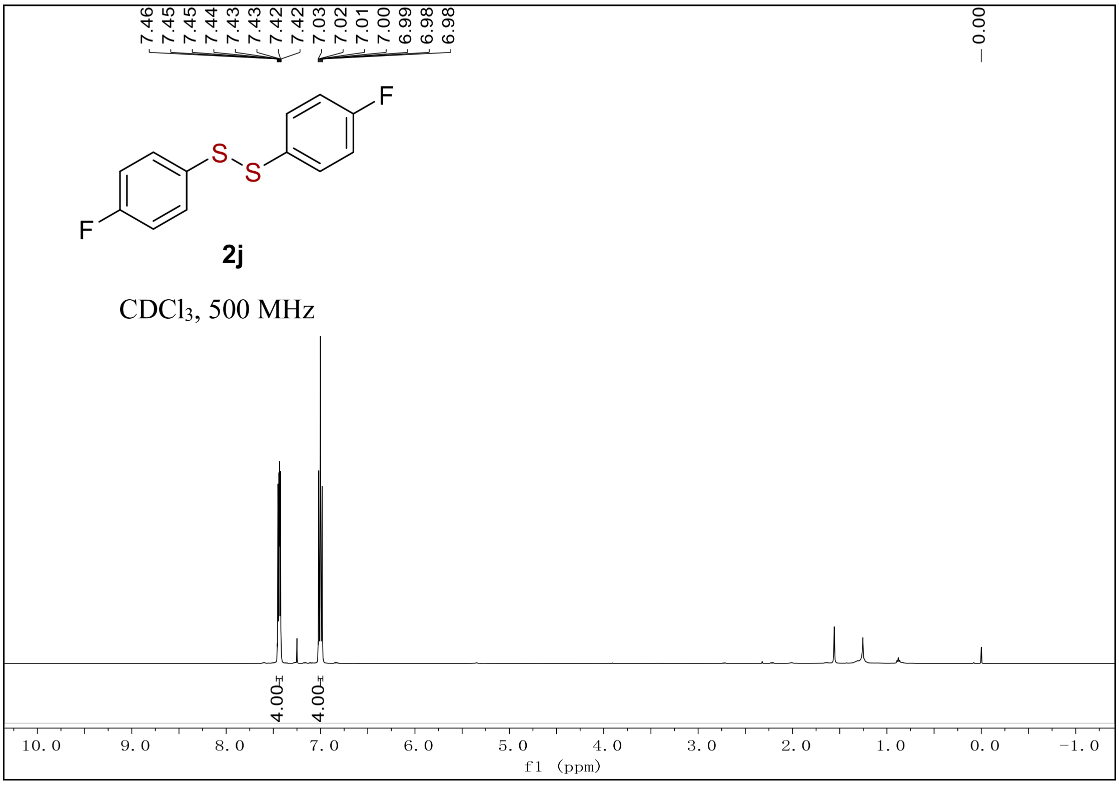
7.46
7.45
7.45
7.44
7.43
7.43
7.42
7.42
7.03
7.02
7.01
7.00
6.99
6.98
6.98
0.00
F
S
S
F
2j
CDCl3, 500 MHz
4.00
4.00
10.0
9.0
8.0
7.0
6.0
5.0
4.0
3.0
2.0
1.0
0.0
-1.0
f1 (ppm)

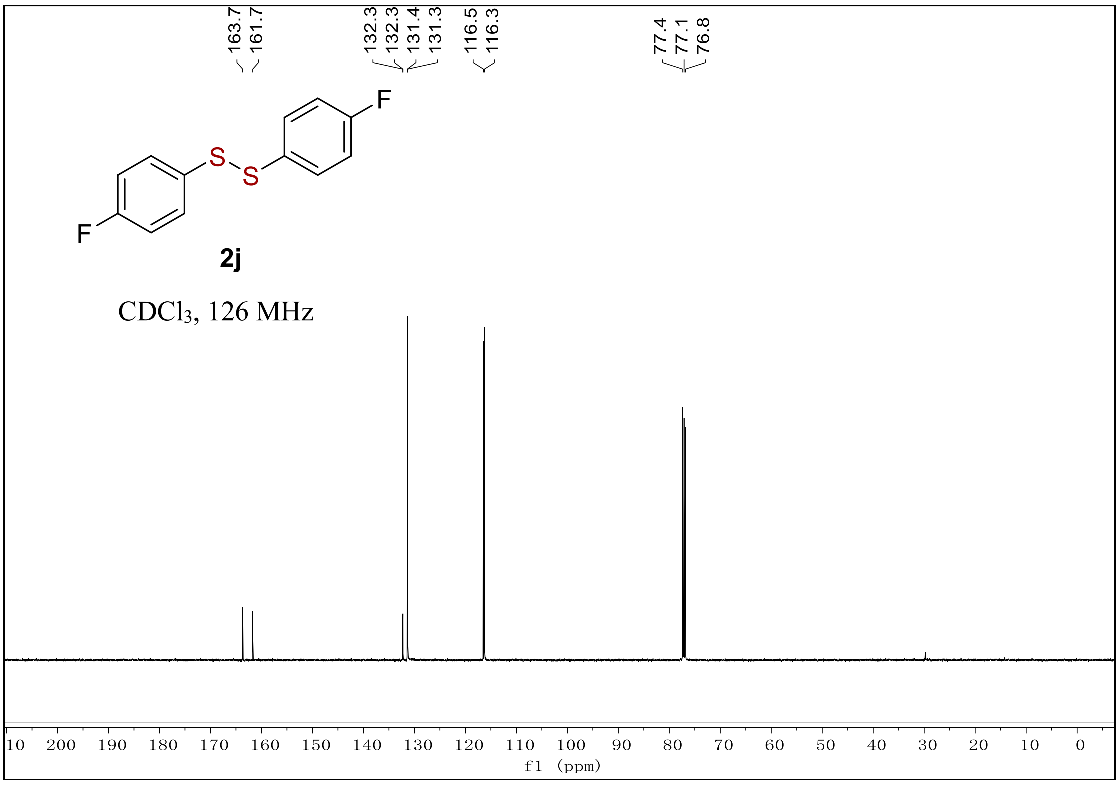
163.7
161.7
132.3
132.3
131.4
131.3
116.5
116.3
77.4
77.1
76.8
F
S
S
F
2j
CDCl3, 126 MHz
10
200
190
180
170
160
150
140
130
120
110
100
90
80
70
60
50
40
30
20
10
0
f1 (ppm)

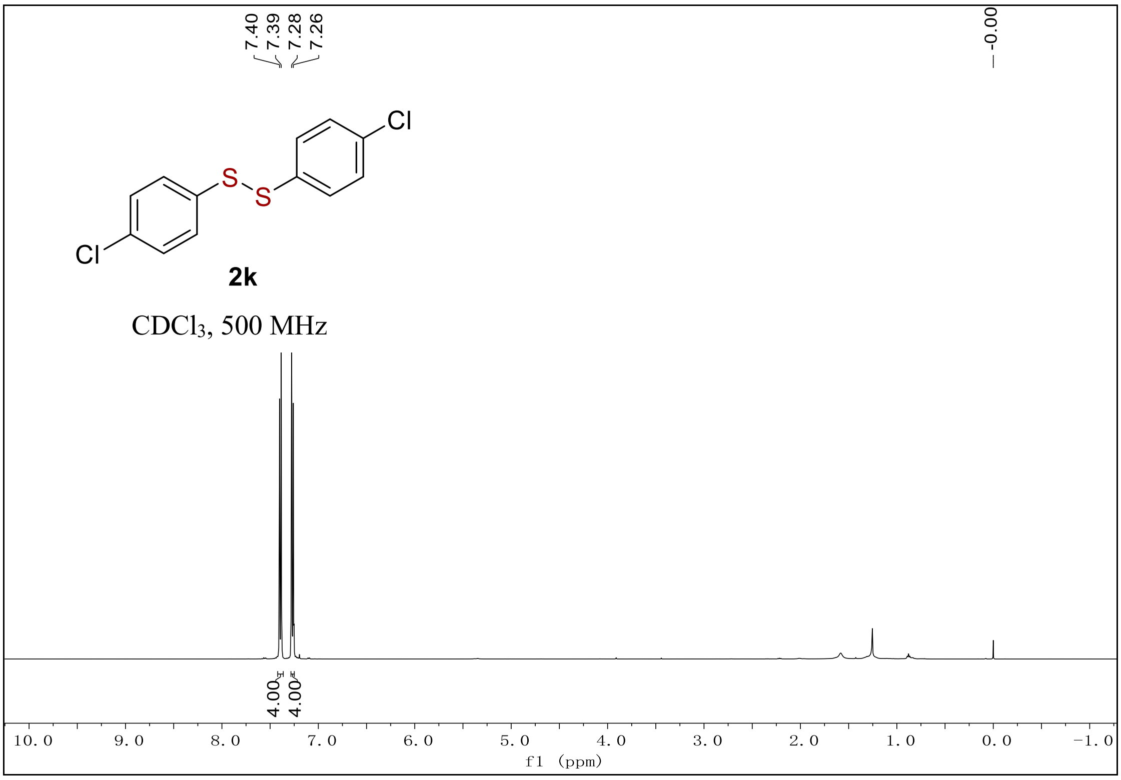
7.40
7.39
7.28
7.26
-0.00
Cl
S
S
Cl
2k
CDCl3, 500 MHz
4.00
4.00
10.0
9.0
8.0
7.0
6.0
5.0
4.0
3.0
2.0
1.0
0.0
-1.0
f1 (ppm)

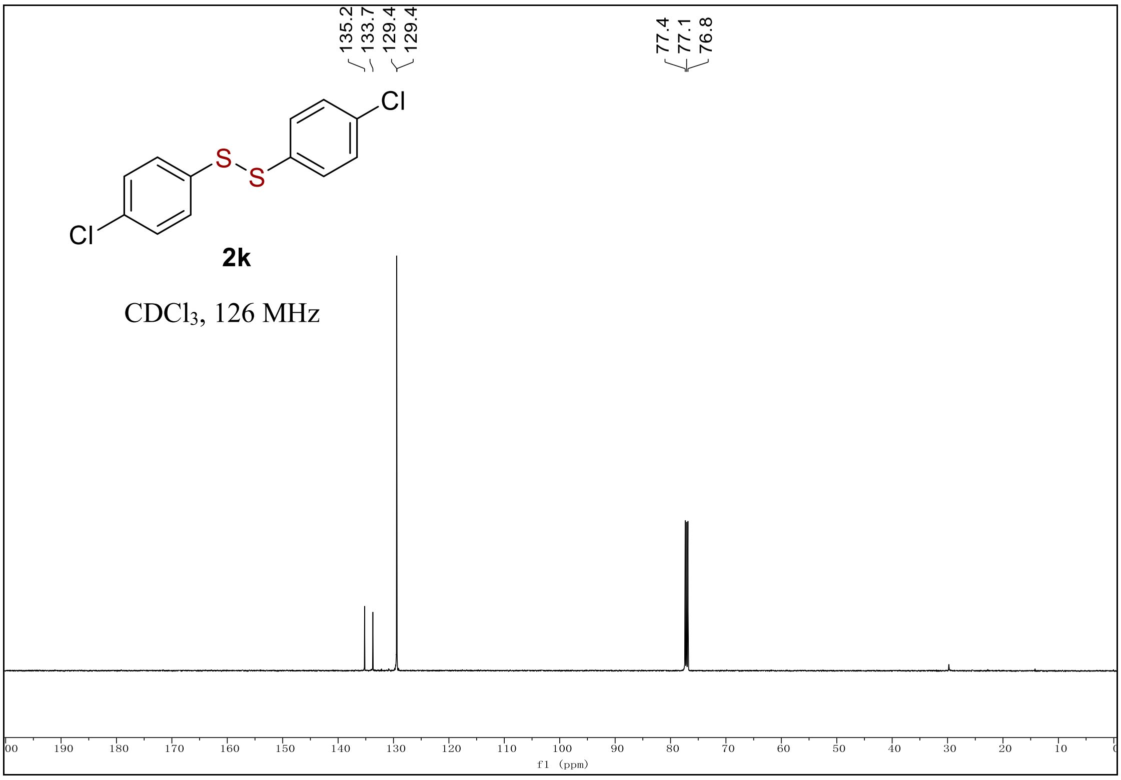
135.2
133.7
129.4
129.4
77.4
77.1
76.8
Cl
S
S
Cl
2k
CDCl3, 126 MHz
190
180
170
160
150
140
130
120
110
100
90
80
70
60
50
40
30
20
10
f1 (ppm)

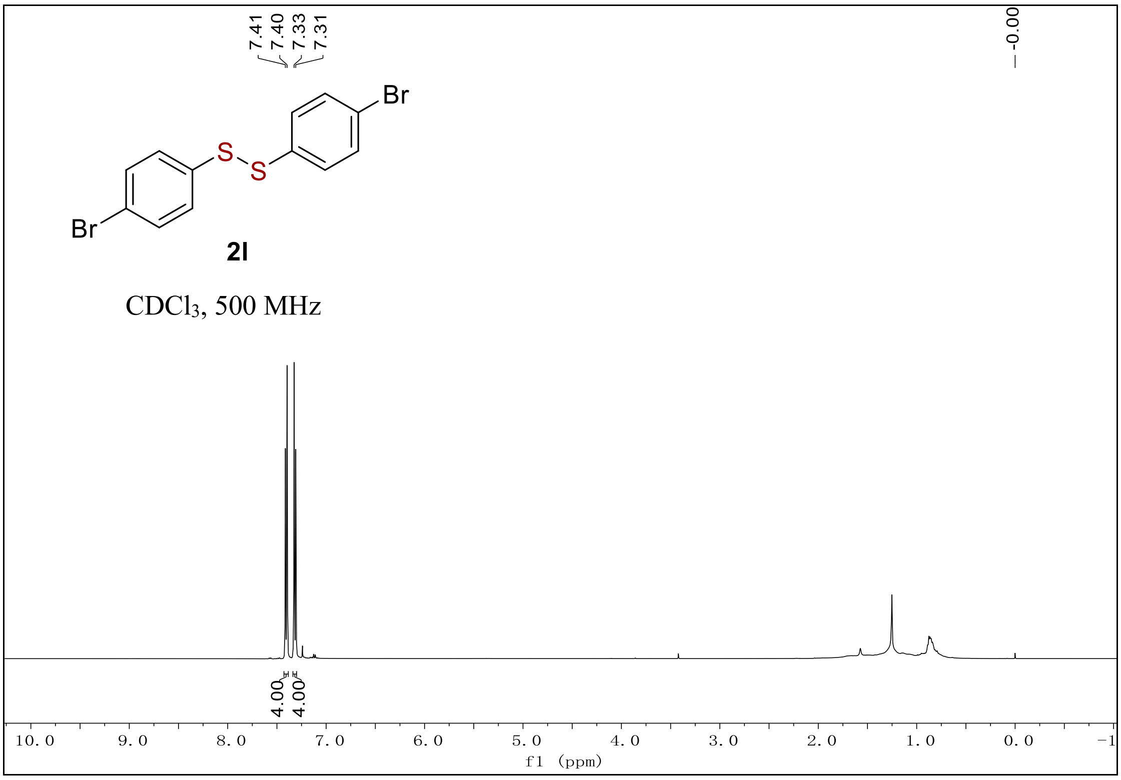
7.41
7.40
7.33
7.31
-0.00
Br
S
S
Br
2l
CDCl3, 500 MHz
4.00
4.00
10.0
9.0
8.0
7.0
6.0
5.0
4.0
3.0
2.0
1.0
0.0
f1 (ppm)

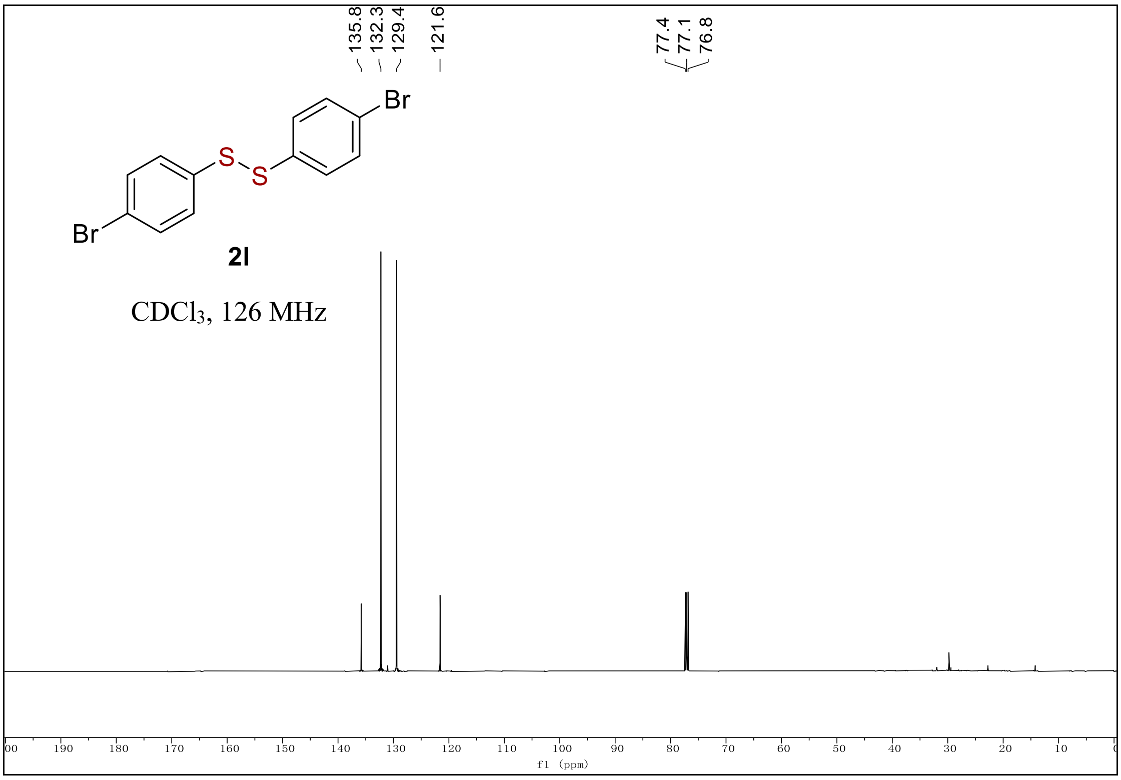
135.8
132.3
129.4
121.6
77.4
77.1
76.8
Br
S
S
Br
2l
CDCl3, 126 MHz
190
180
170
160
150
140
130
120
110
100
90
80
70
60
50
40
30
20
10
f1 (ppm)

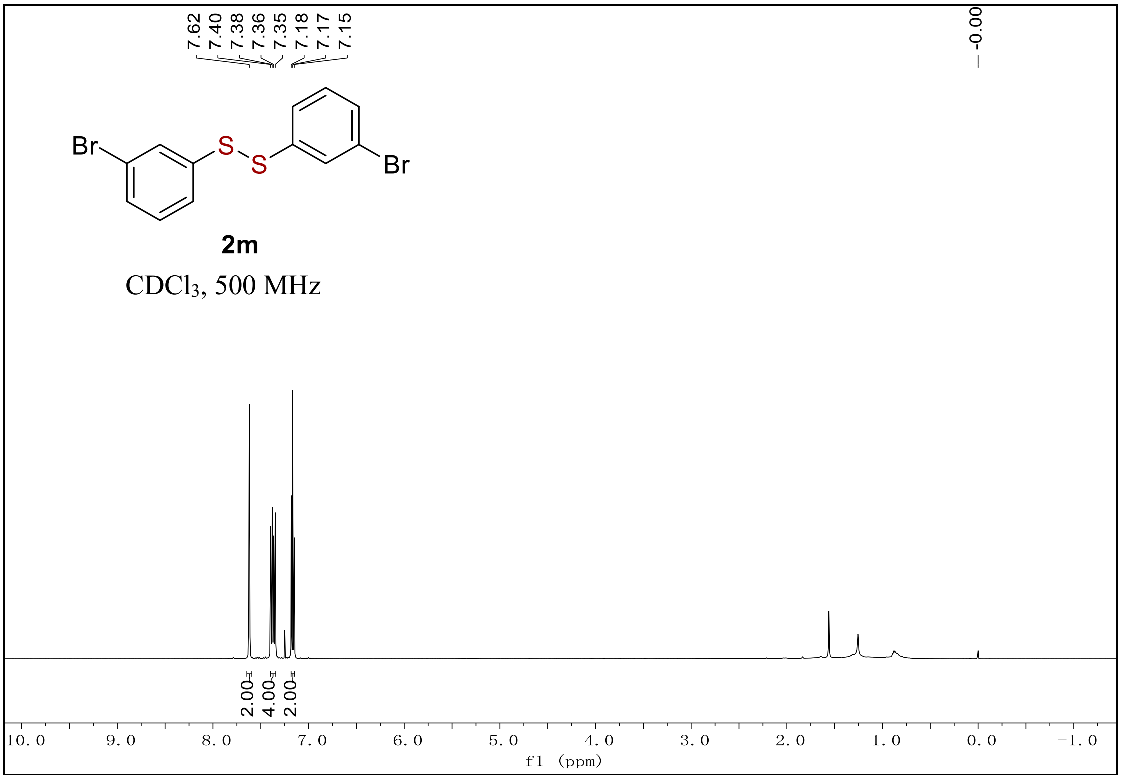
7.62
7.40
7.38
7.36
7.35
7.18
7.17
7.15
-0.00
Br
S
S
Br
2m
CDCl3, 500 MHz
2.00
4.00
2.00
10.0
9.0
8.0
7.0
6.0
5.0
4.0
3.0
2.0
1.0
0.0
-1.0
f1 (ppm)

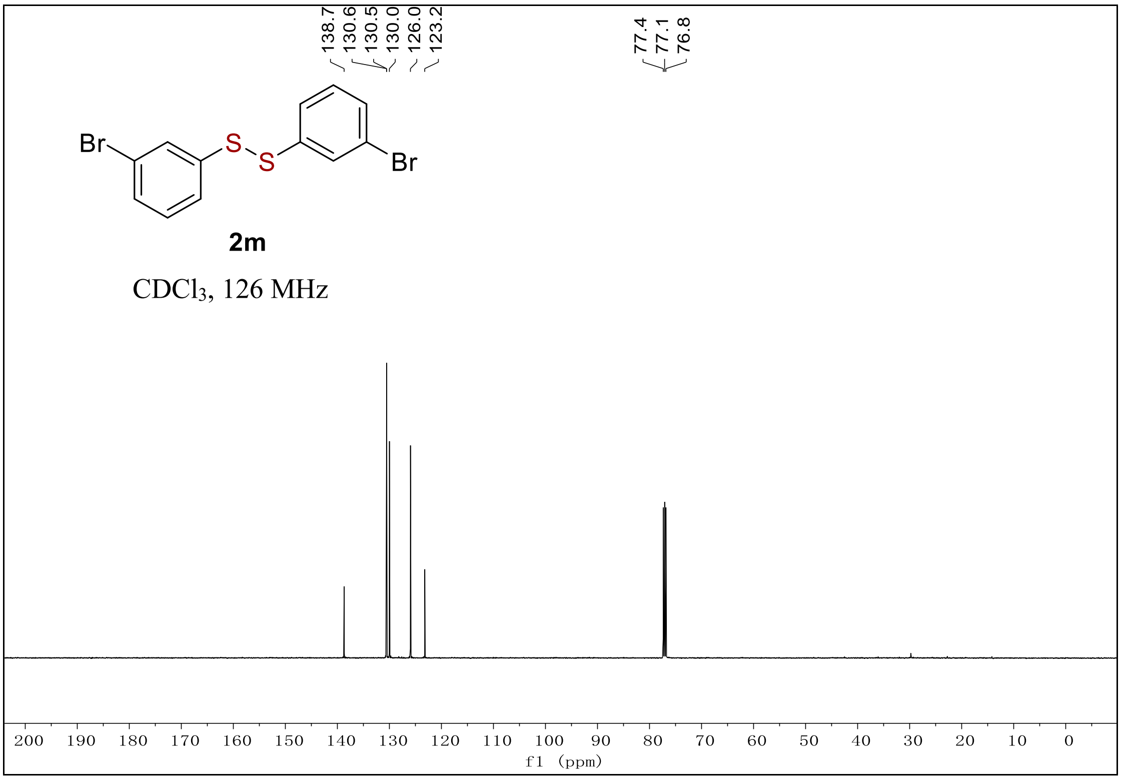
138.7
130.6
130.5
130.0
126.0
123.2
77.4
77.1
76.8
Br
S
S
Br
2m
CDCl3, 126 MHz
200
190
180
170
160
150
140
130
120
110
100
90
80
70
60
50
40
30
20
10
0
f1 (ppm)

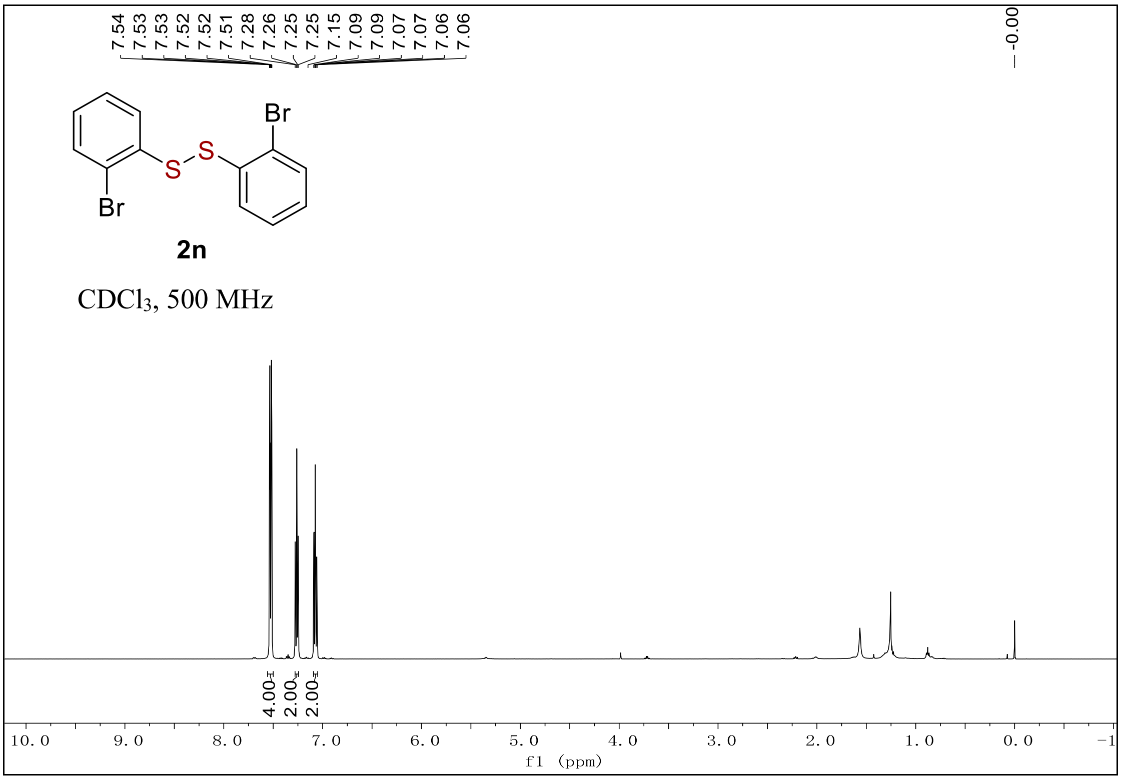
7.54
7.53
7.53
7.52
7.52
7.51
7.28
7.26
7.25
7.25
7.15
7.09
7.09
7.07
7.07
7.06
7.06
-0.00
Br
S
S
Br
2n
CDCl3, 500 MHz
4.00
2.00
2.00
10.0 9.0 8.0 7.0 6.0 5.0 4.0 3.0 2.0 1.0 0.0 -1
f1 (ppm)

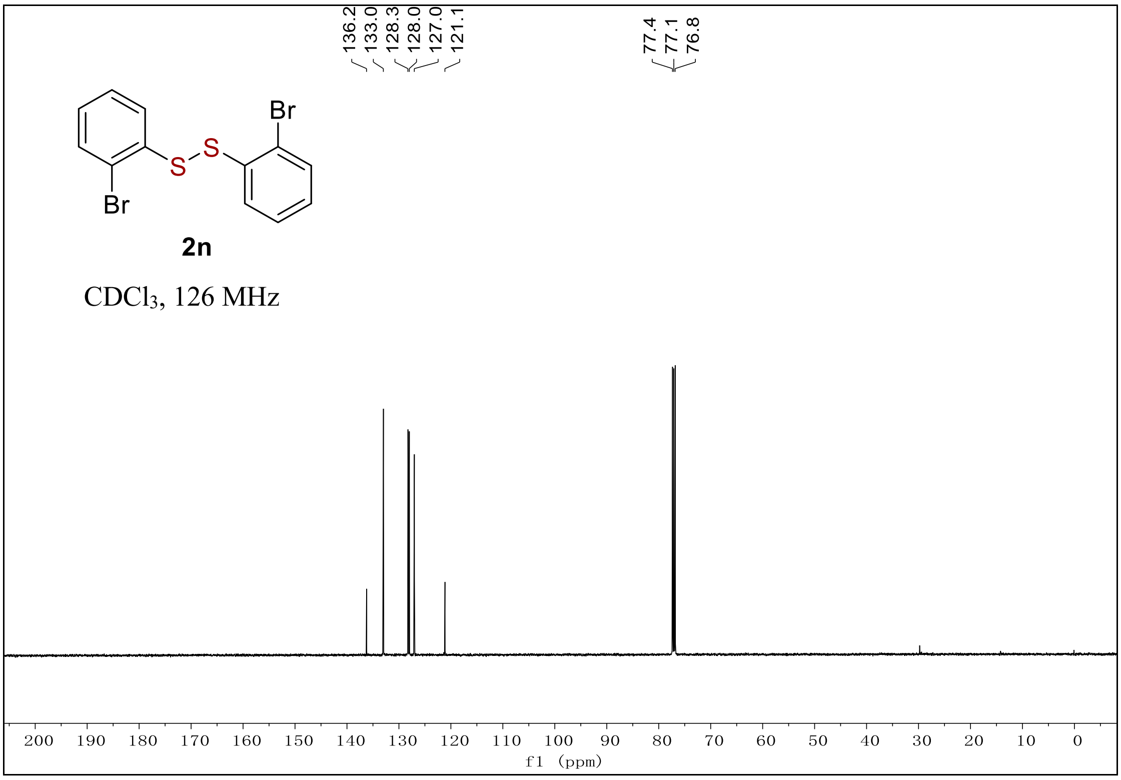
136.2
133.0
128.3
128.0
127.0
121.1
77.4
77.1
76.8
Br
S
S
Br
2n
CDCl3, 126 MHz
200 190 180 170 160 150 140 130 120 110 100 90 80 70 60 50 40 30 20 10 0
f1 (ppm)

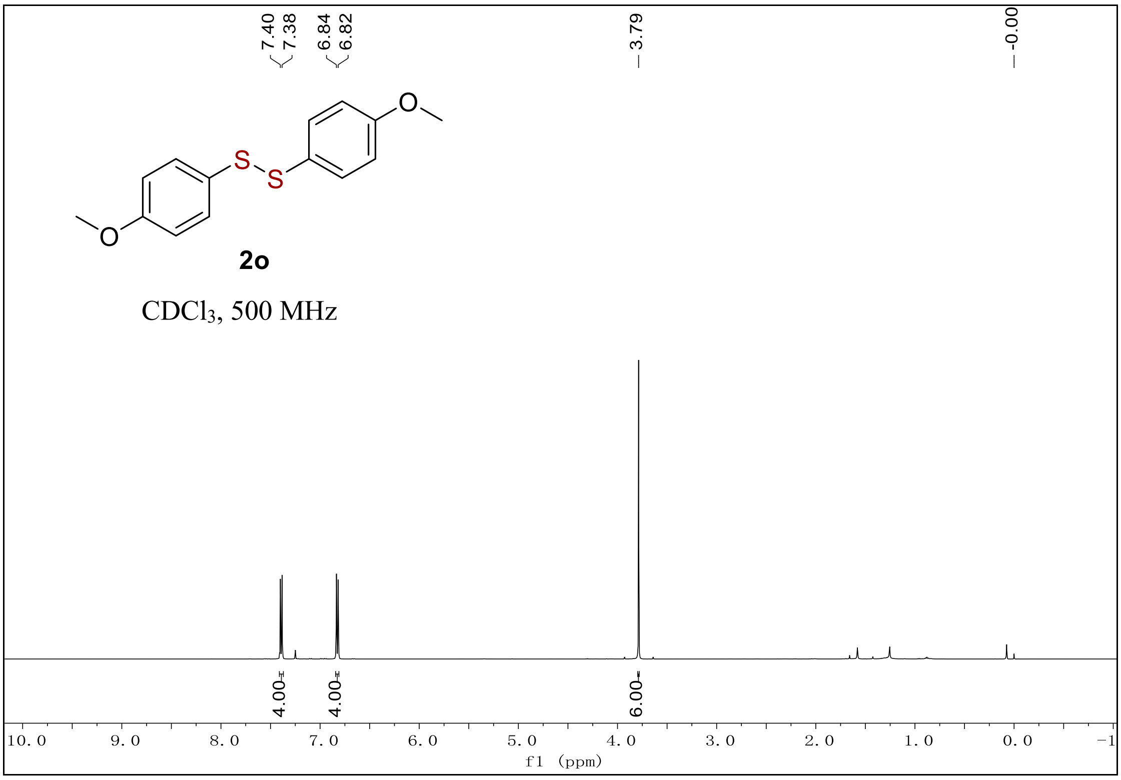
7.40
7.38
6.84
6.82
3.79
-0.00
S
S
O
O
2o
CDCl3, 500 MHz
4.00
4.00
6.00
10.0
9.0
8.0
7.0
6.0
5.0
4.0
3.0
2.0
1.0
0.0
-1
f1 (ppm)

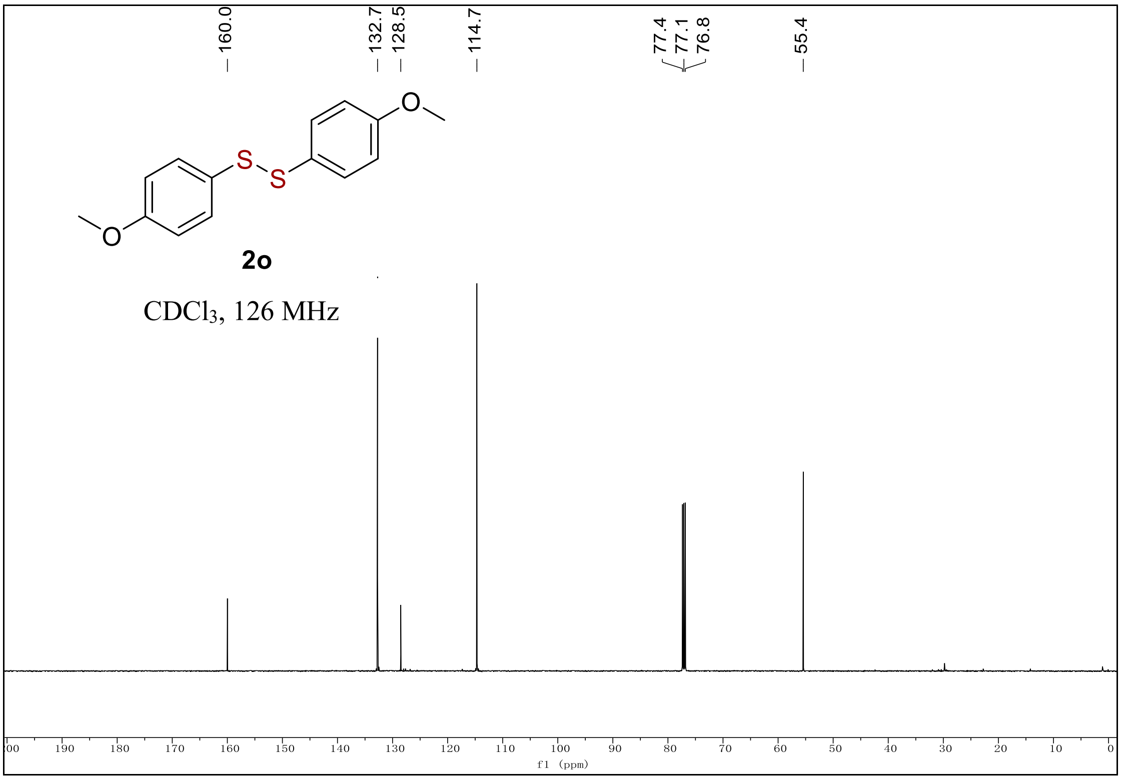
160.0
132.7
128.5
114.7
77.4
77.1
76.8
55.4
S
S
O
O
2o
CDCl3, 126 MHz
190
180
170
160
150
140
130
120
110
100
90
80
70
60
50
40
30
20
10
0
f1 (ppm)

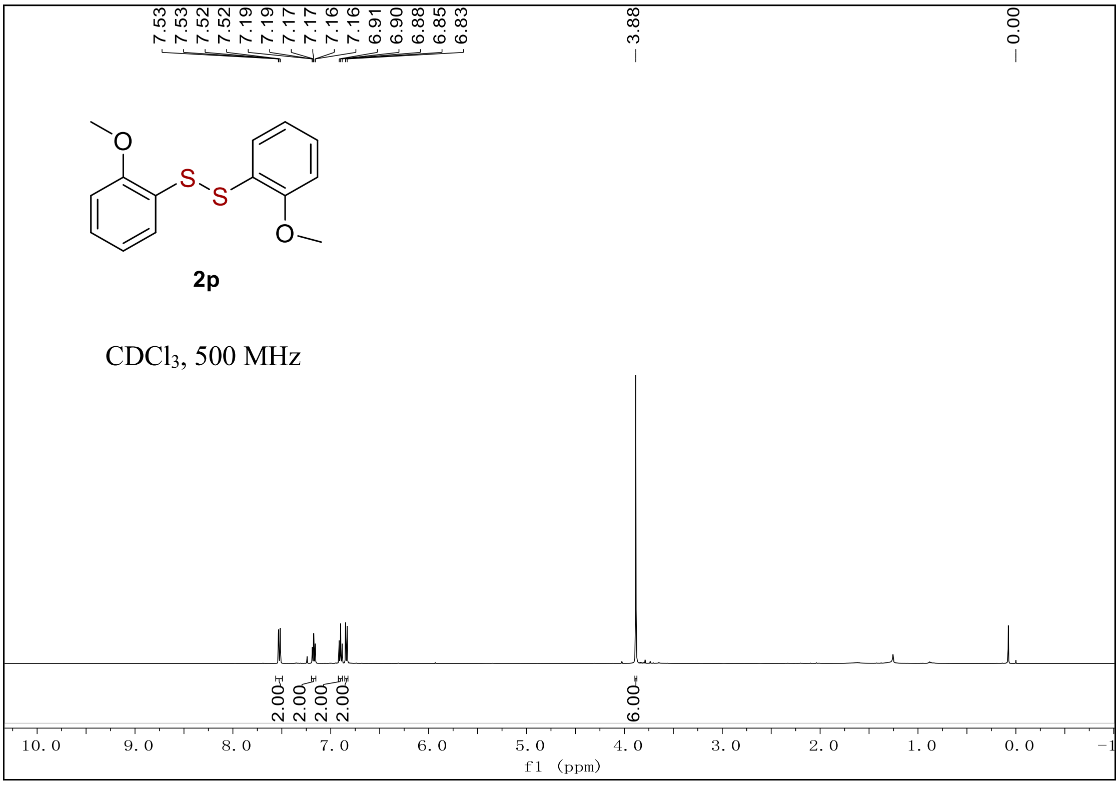
7.53
7.53
7.52
7.52
7.19
7.19
7.17
7.17
7.16
7.16
6.91
6.90
6.88
6.85
6.83
3.88
0.00
2p
CDCl3, 500 MHz
2.00
2.00
2.00
2.00
6.00
10.0
9.0
8.0
7.0
6.0
5.0
4.0
3.0
2.0
1.0
0.0
f1 (ppm)

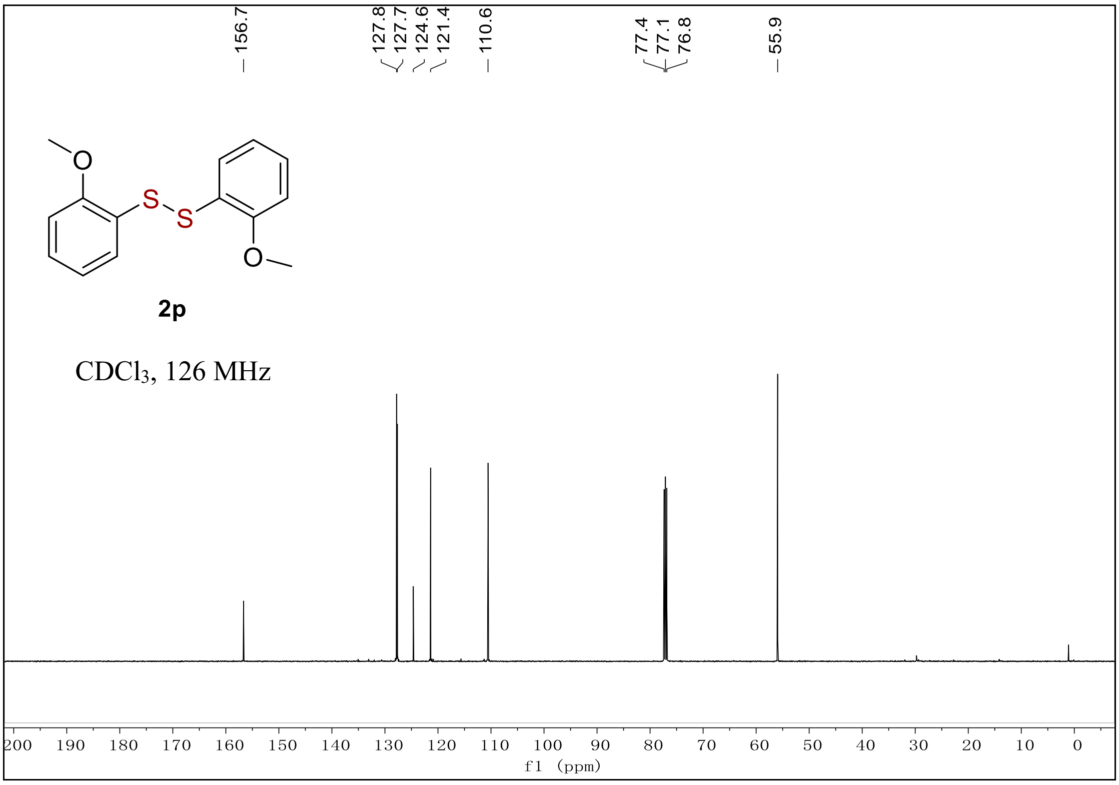
156.7
127.8
127.7
124.6
121.4
110.6
77.4
77.1
76.8
55.9
2p
CDCl3, 126 MHz
200
190
180
170
160
150
140
130
120
110
100
90
80
70
60
50
40
30
20
10
0
f1 (ppm)

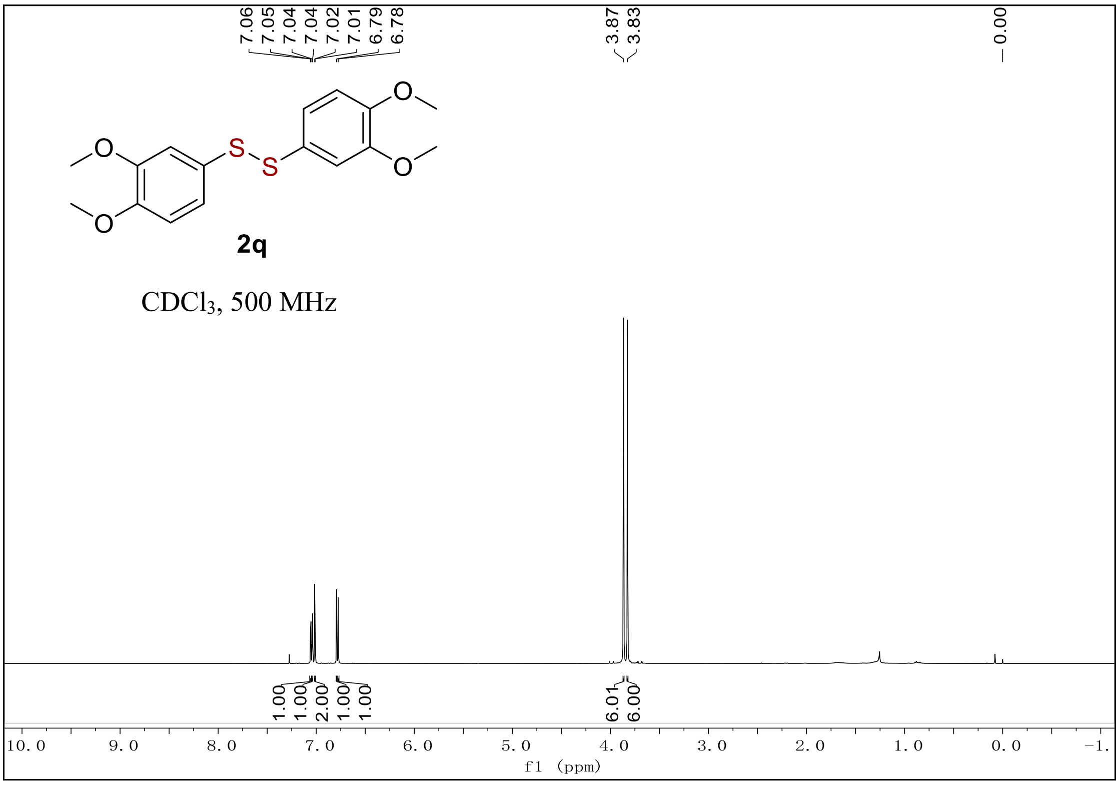
7.06
7.05
7.04
7.04
7.02
7.01
6.79
6.78
3.87
3.83
0.00
2q
CDCl3, 500 MHz
1.00
1.00
2.00
1.00
1.00
6.01
6.00
10.0 9.0 8.0 7.0 6.0 5.0 4.0 3.0 2.0 1.0 0.0 -1.
f1 (ppm)

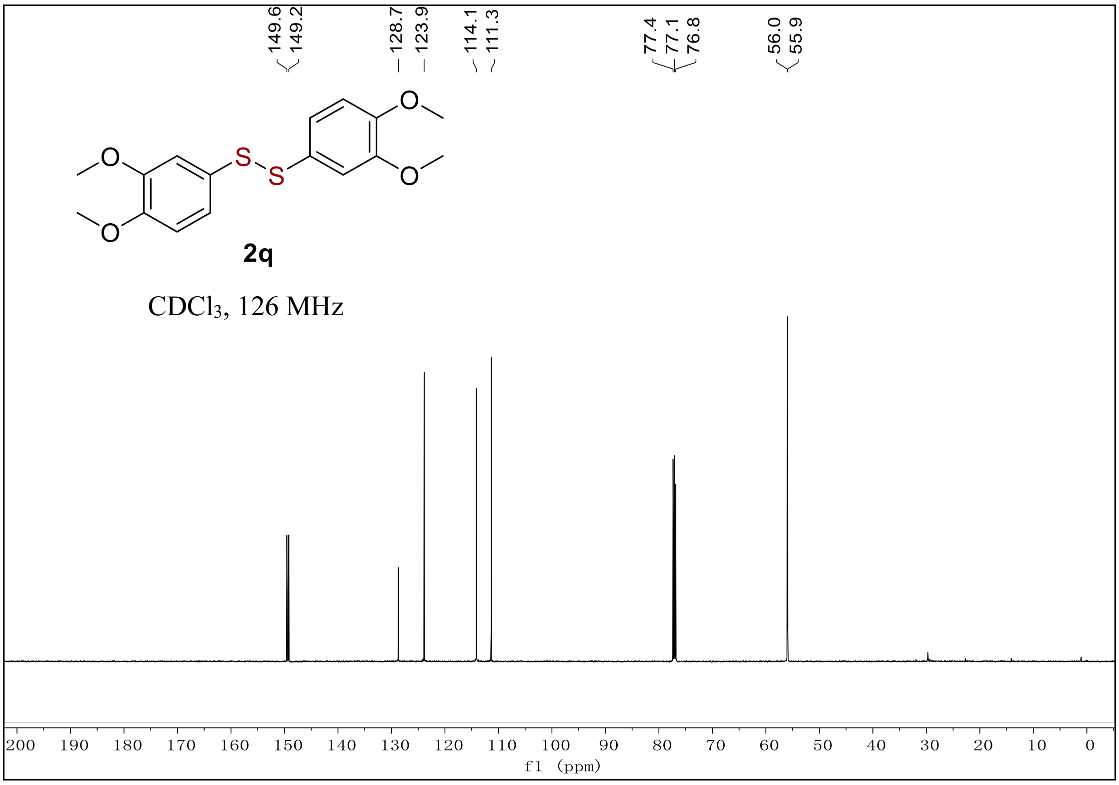
149.6
149.2
128.7
123.9
114.1
111.3
77.4
77.1
76.8
56.0
55.9
2q
CDCl3, 126 MHz
200 190 180 170 160 150 140 130 120 110 100 90 80 70 60 50 40 30 20 10 0
f1 (ppm)

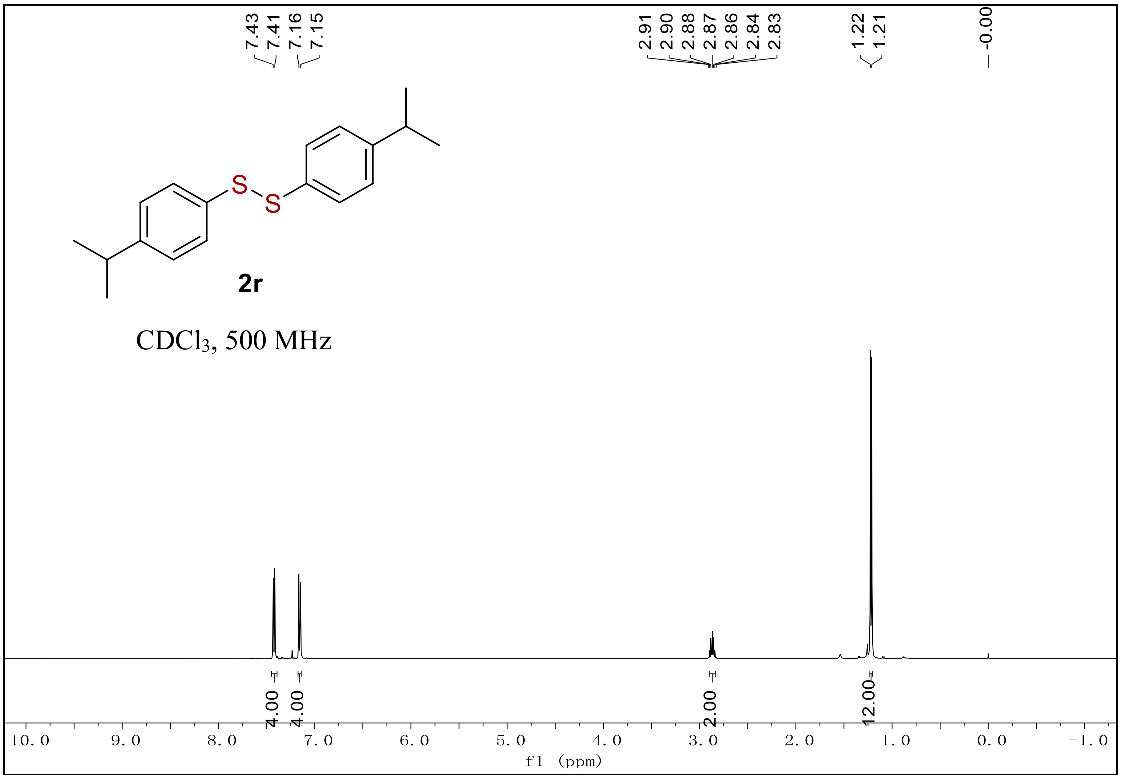
7.43
7.41
7.16
7.15
2.91
2.90
2.88
2.87
2.86
2.84
2.83
1.22
1.21
-0.00
2r
CDCl3, 500 MHz
4.00
4.00
2.00
12.00
10.0 9.0 8.0 7.0 6.0 5.0 4.0 3.0 2.0 1.0 0.0 -1.0
f1 (ppm)

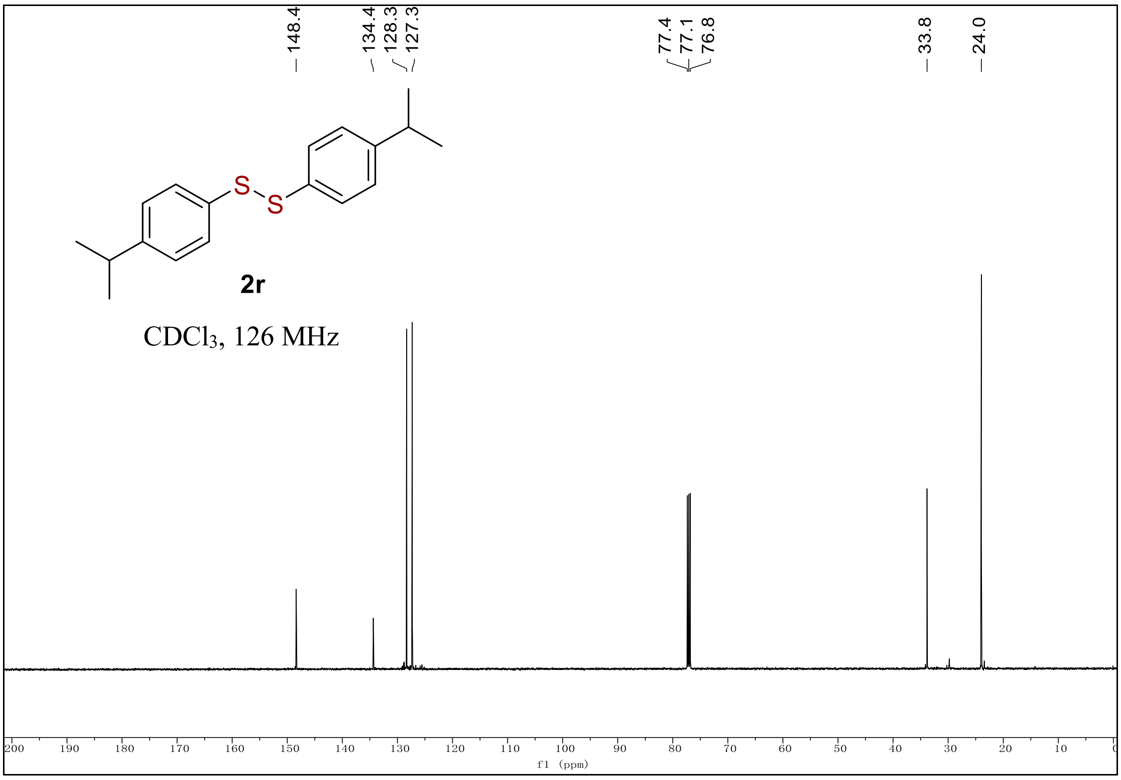
148.4
134.4
128.3
127.3
77.4
77.1
76.8
33.8
24.0
2r
CDCl3, 126 MHz
200 190 180 170 160 150 140 130 120 110 100 90 80 70 60 50 40 30 20 10
f1 (ppm)

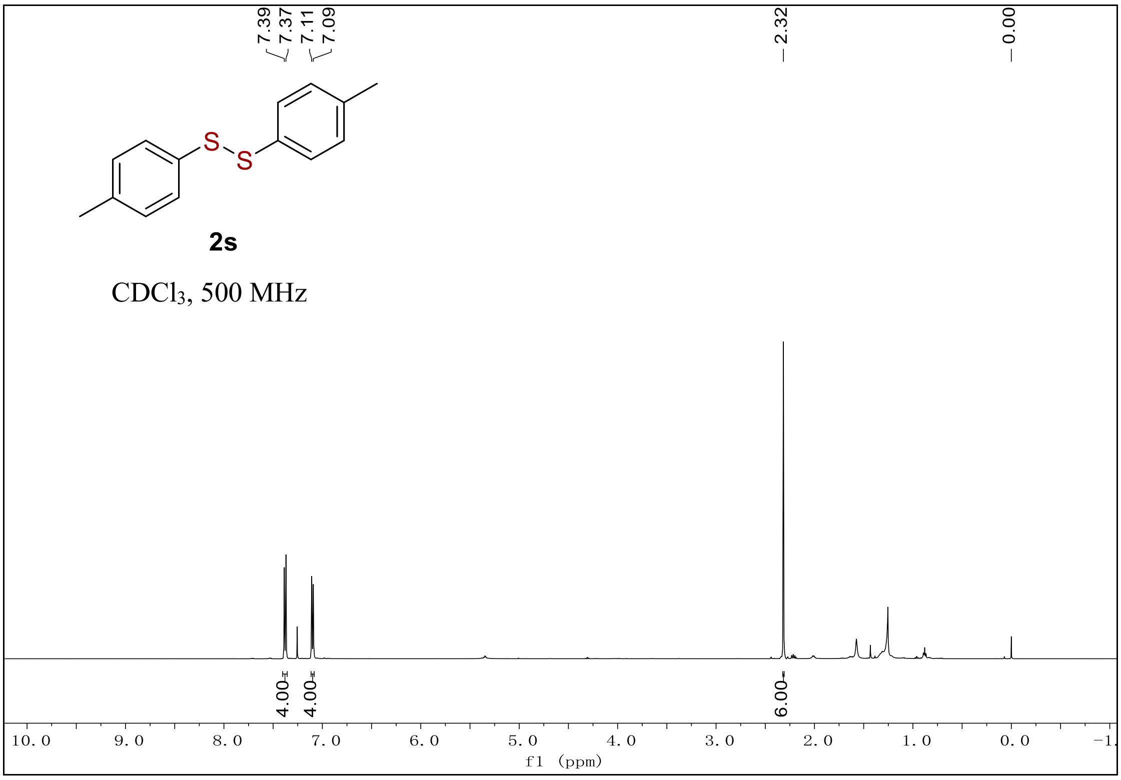
7.39
7.37
7.11
7.09
2.32
0.00
2s
CDCl3, 500 MHz
4.00
4.00
6.00
10.0
9.0
8.0
7.0
6.0
5.0
4.0
3.0
2.0
1.0
0.0
f1 (ppm)

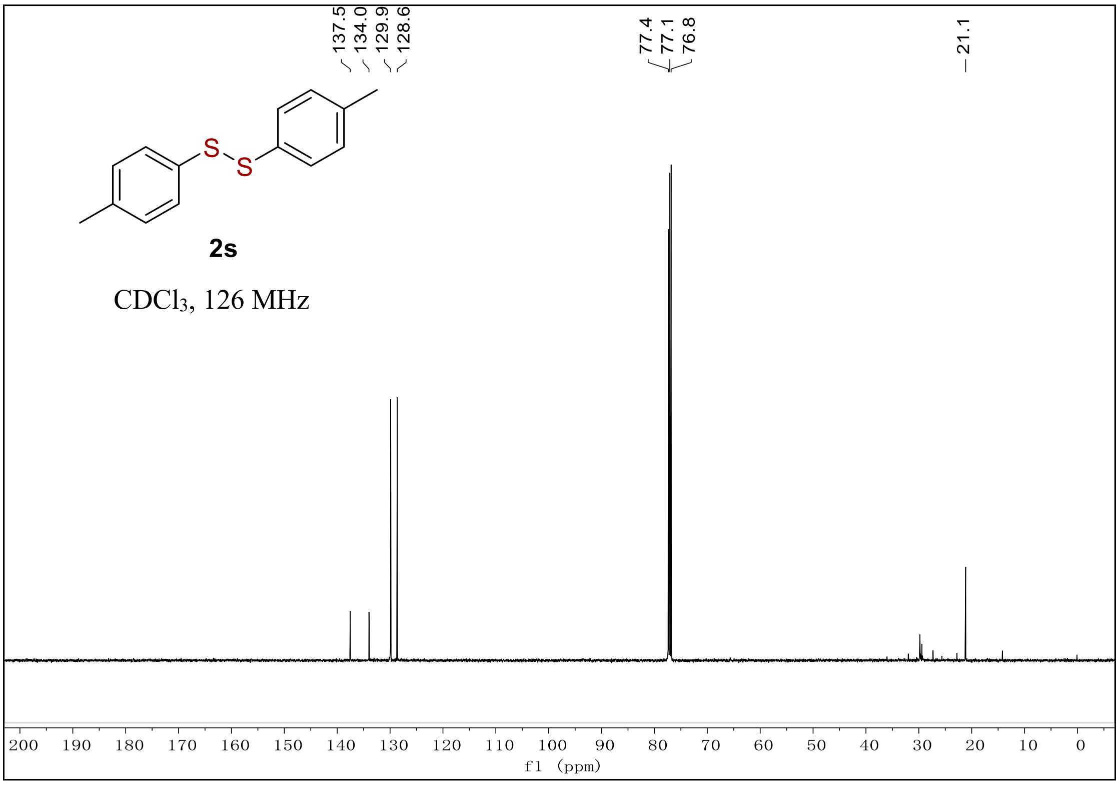
137.5
134.0
129.9
128.6
77.4
77.1
76.8
21.1
2s
CDCl3, 126 MHz
200
190
180
170
160
150
140
130
120
110
100
90
80
70
60
50
40
30
20
10
0
f1 (ppm)

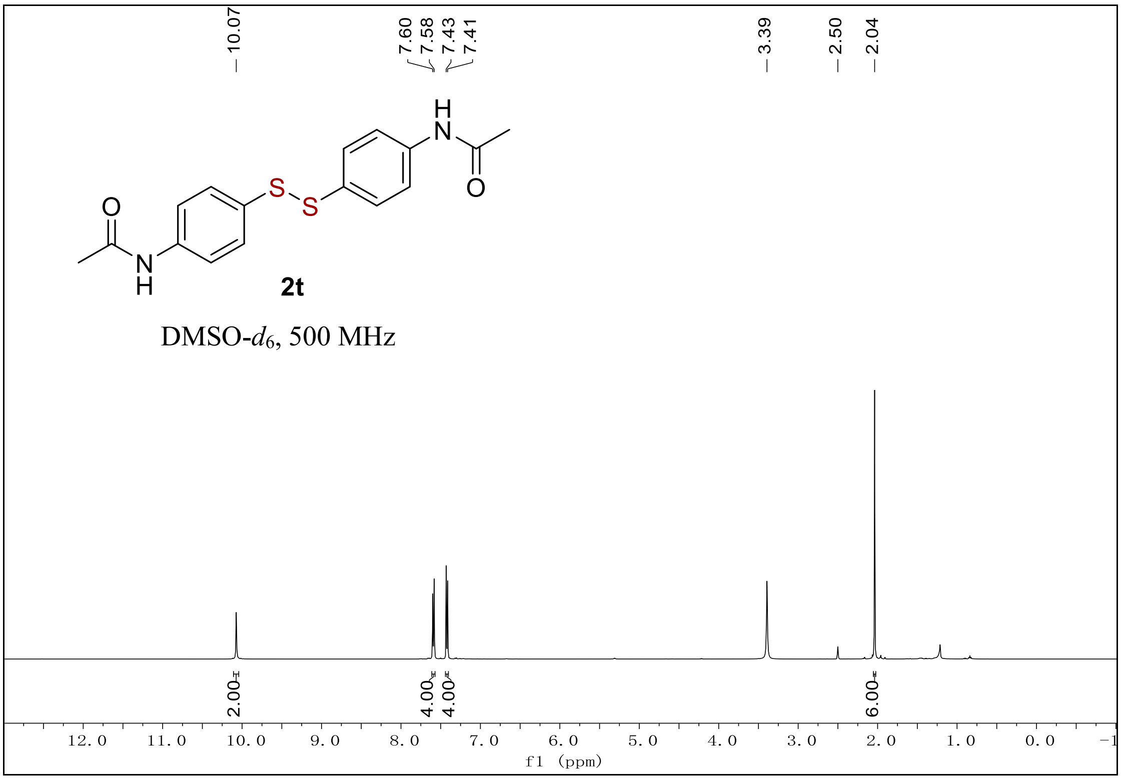
10.07
7.60
7.58
7.43
7.41
3.39
2.50
2.04
2t
DMSO-d_6, 500 MHz
2.00
4.00
4.00
6.00
12.0 11.0 10.0 9.0 8.0 7.0 6.0 5.0 4.0 3.0 2.0 1.0 0.0
f1 (ppm)

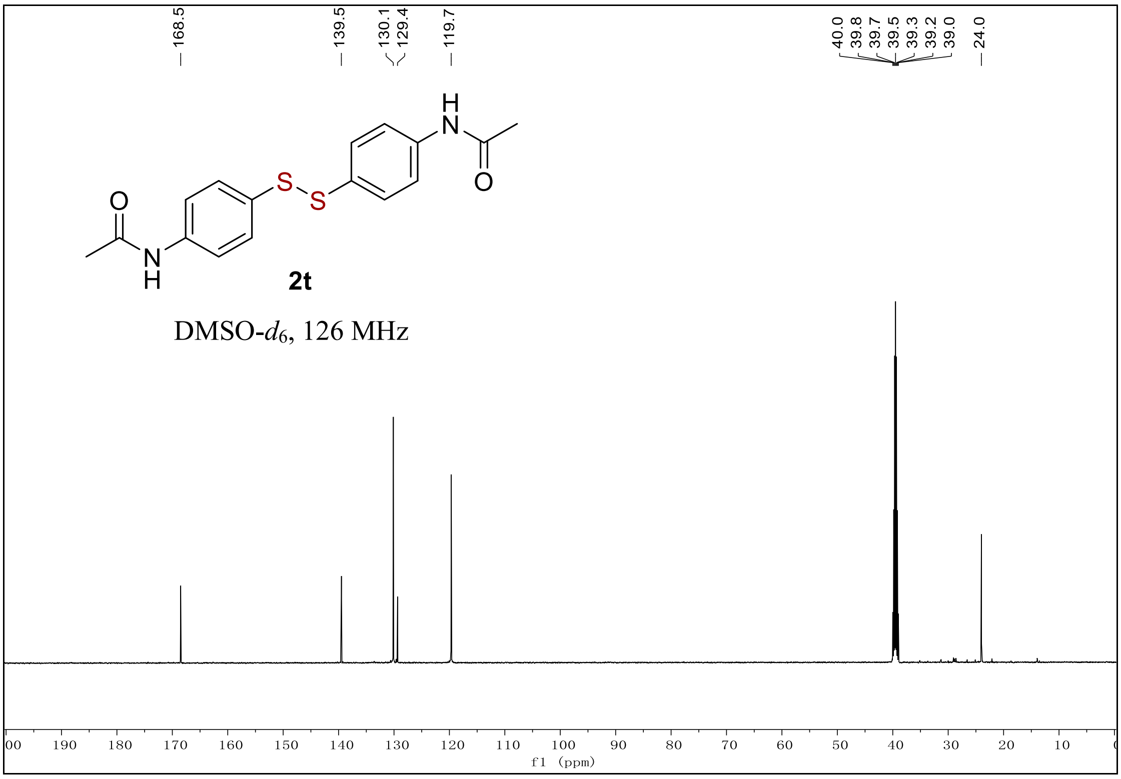
168.5
139.5
130.1
129.4
119.7
40.0
39.8
39.7
39.5
39.3
39.2
39.0
24.0
2t
DMSO-d_6, 126 MHz
190 180 170 160 150 140 130 120 110 100 90 80 70 60 50 40 30 20 10
f1 (ppm)

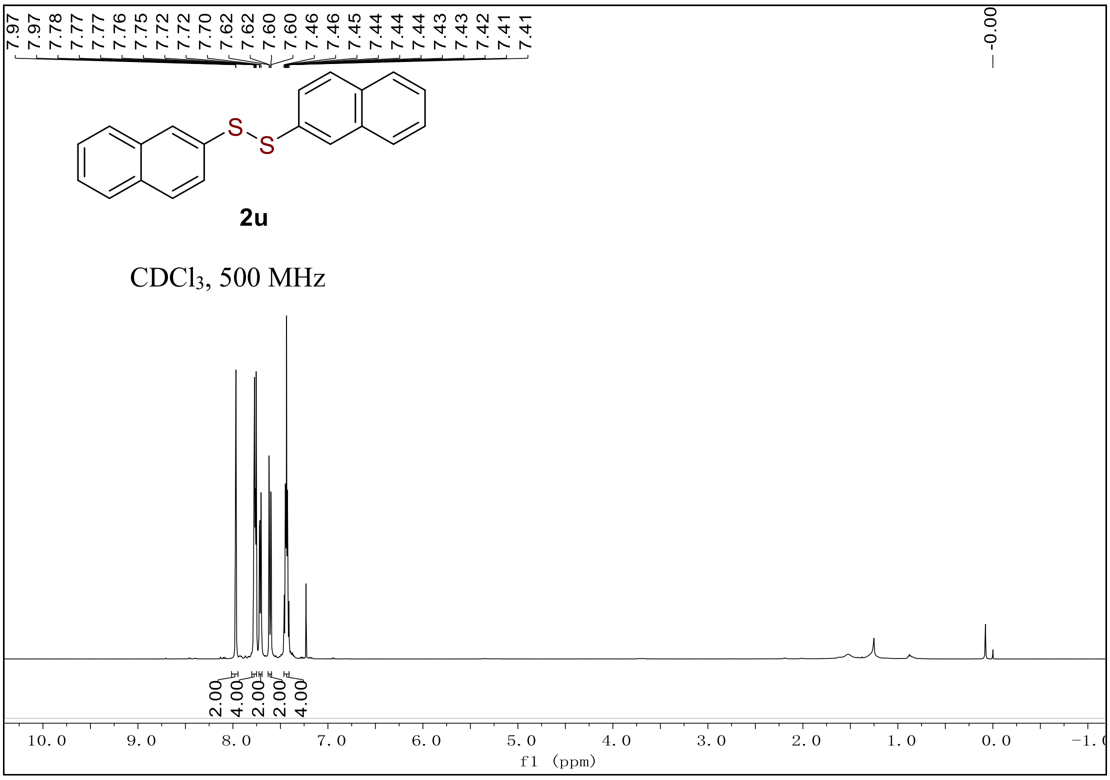
7.97
7.97
7.78
7.77
7.77
7.76
7.75
7.72
7.72
7.70
7.62
7.62
7.60
7.60
7.46
7.46
7.45
7.44
7.44
7.44
7.43
7.43
7.42
7.41
7.41
-0.00
S
S
2u
CDCl3, 500 MHz
2.00
4.00
2.00
2.00
4.00
10.0
9.0
8.0
7.0
6.0
5.0
4.0
3.0
2.0
1.0
0.0
f1 (ppm)

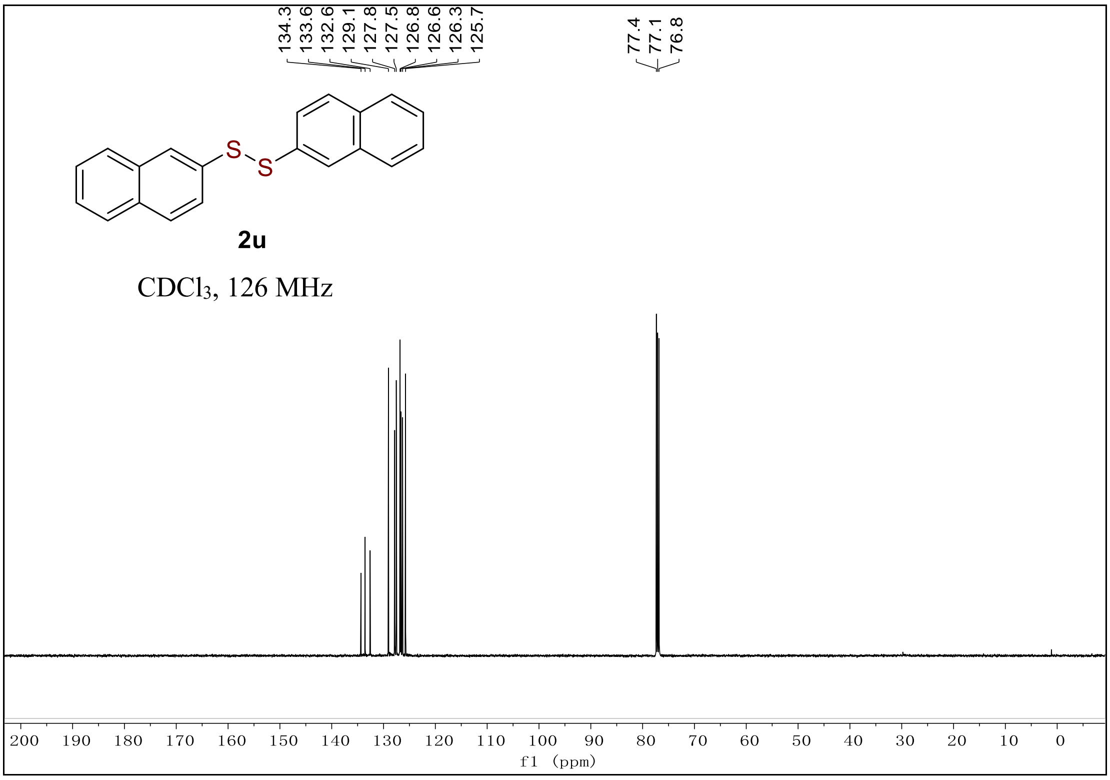
134.3
133.6
132.6
129.1
127.8
127.5
126.8
126.6
126.3
125.7
77.4
77.1
76.8
S
S
2u
CDCl3, 126 MHz
200
190
180
170
160
150
140
130
120
110
100
90
80
70
60
50
40
30
20
10
0
f1 (ppm)

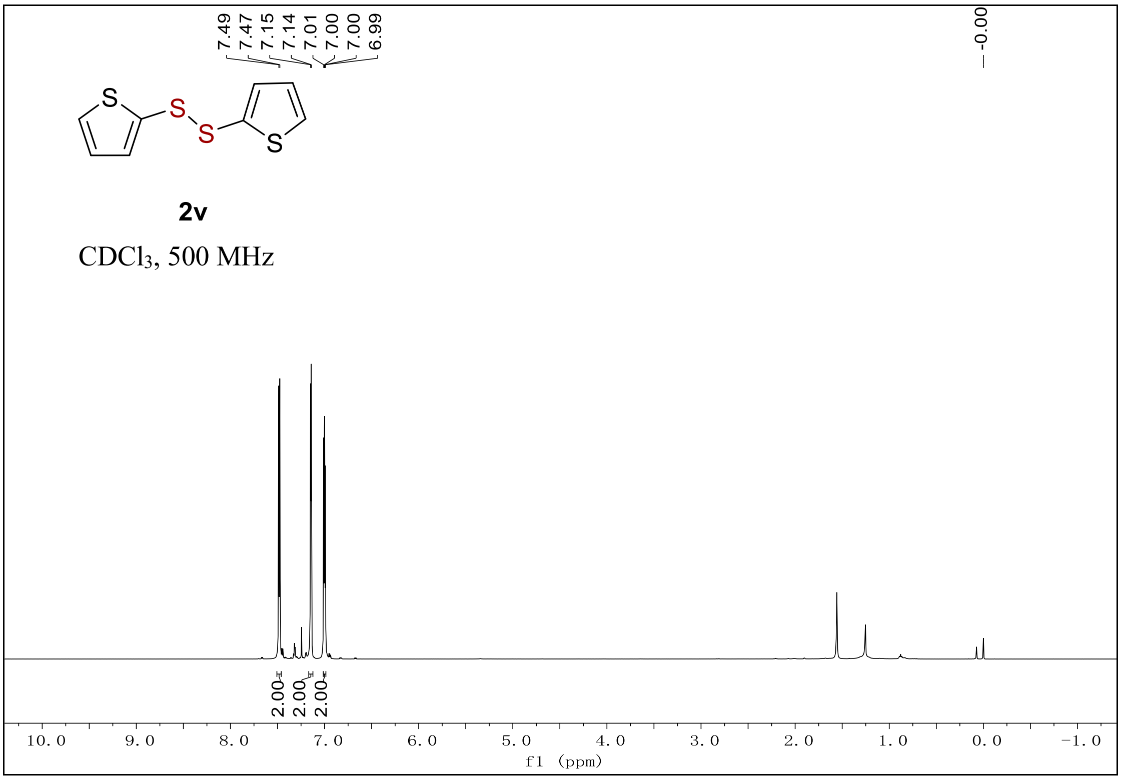
7.49
7.47
7.15
7.14
7.01
7.00
7.00
6.99
-0.00
S
S
S
S
2v
CDCl3, 500 MHz
2.00
2.00
2.00
10.0
9.0
8.0
7.0
6.0
5.0
4.0
3.0
2.0
1.0
0.0
-1.0
f1 (ppm)

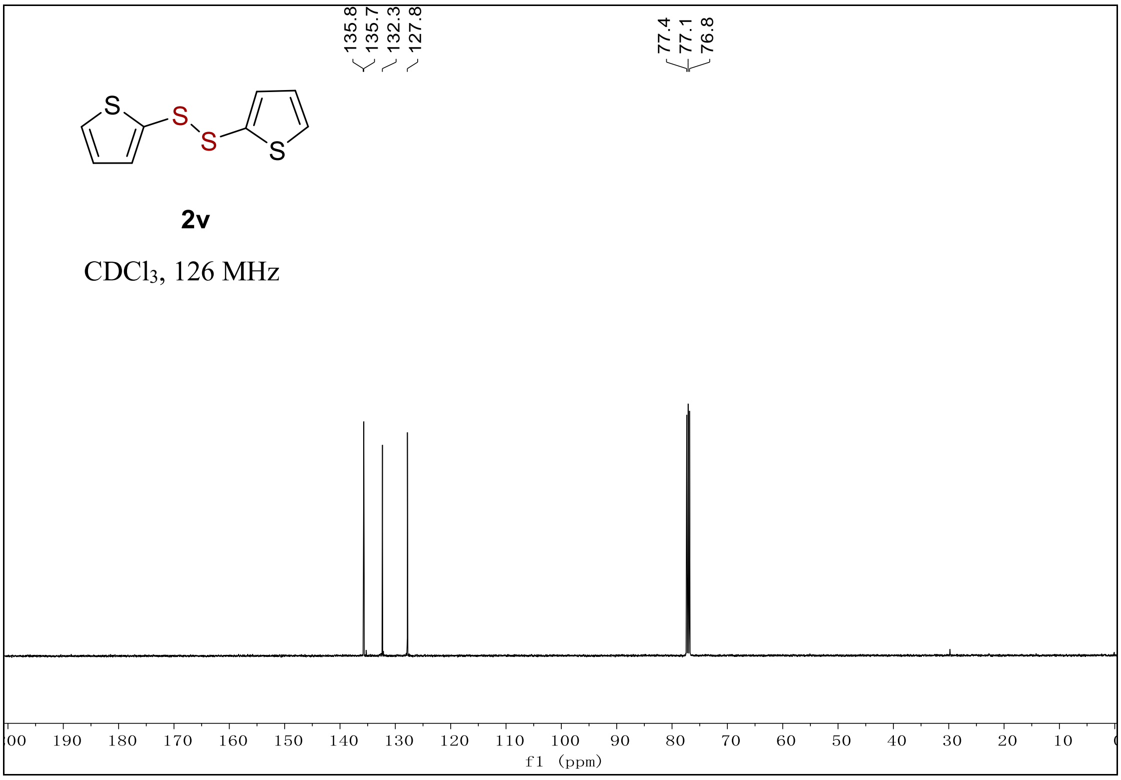
135.8
135.7
132.3
127.8
77.4
77.1
76.8
S
S
S
S
2v
CDCl3, 126 MHz
190
180
170
160
150
140
130
120
110
100
90
80
70
60
50
40
30
20
10
f1 (ppm)

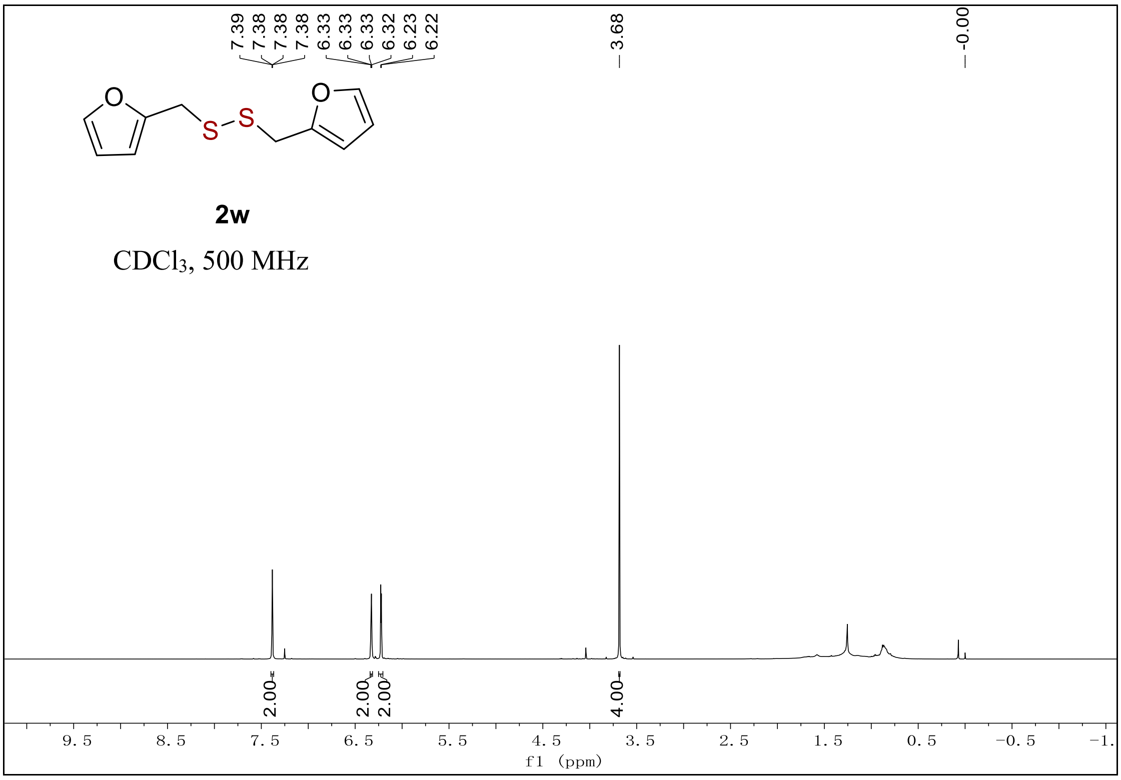
7.39
7.38
7.38
7.38
6.33
6.33
6.33
6.32
6.23
6.22
3.68
-0.00
2w
CDCl3, 500 MHz
2.00
2.00
2.00
4.00
9.5
8.5
7.5
6.5
5.5
4.5
3.5
2.5
1.5
0.5
-0.5
f1 (ppm)

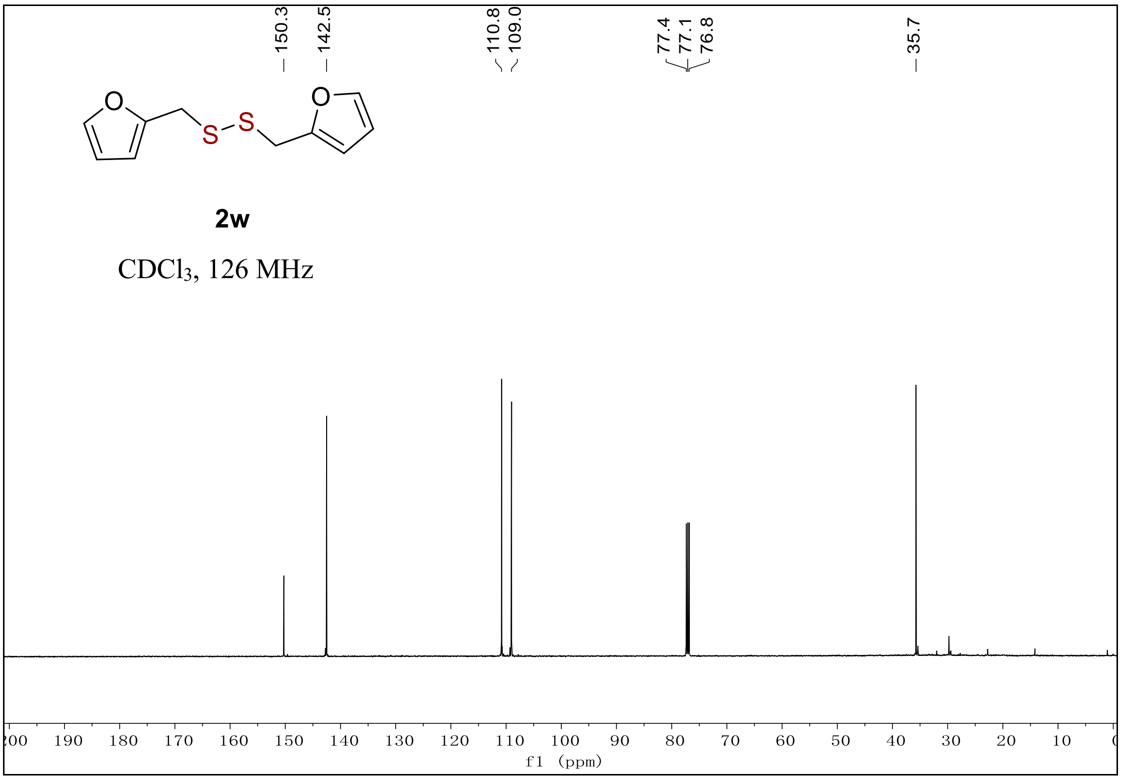
150.3
142.5
110.8
109.0
77.4
77.1
76.8
35.7
2w
CDCl3, 126 MHz
190
180
170
160
150
140
130
120
110
100
90
80
70
60
50
40
30
20
10
f1 (ppm)

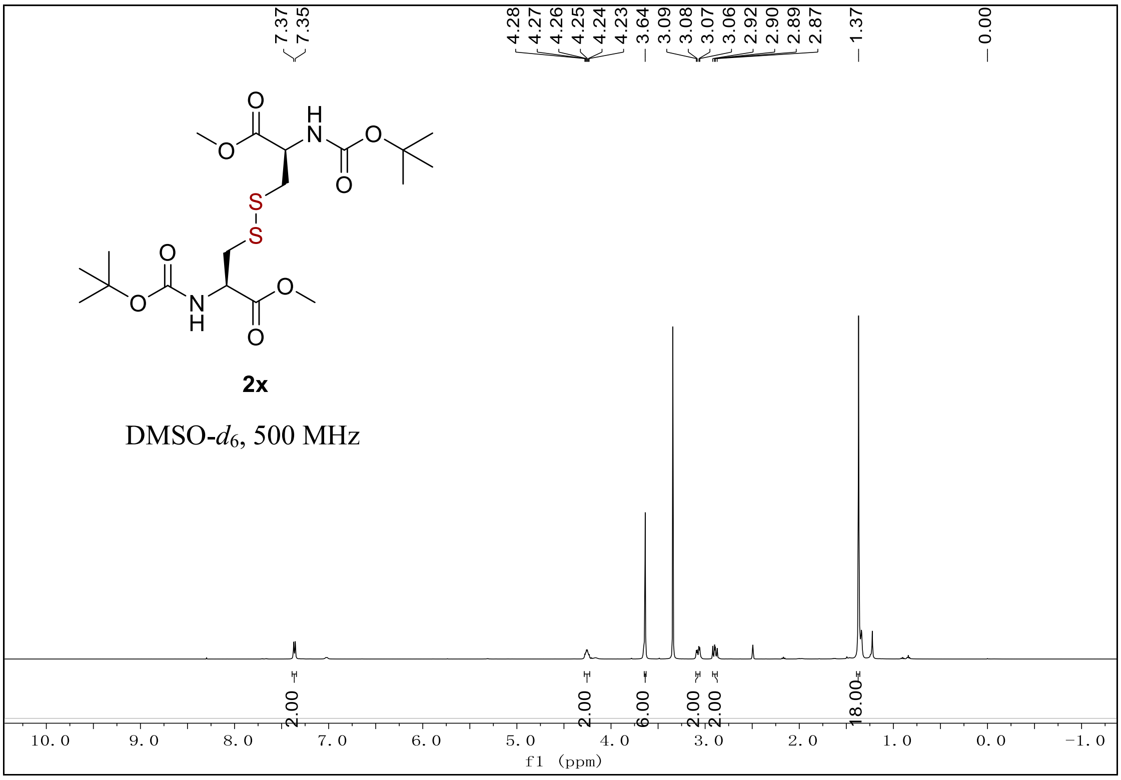
7.37
7.35
4.28
4.27
4.26
4.25
4.24
4.23
3.64
3.09
3.08
3.07
3.06
2.92
2.90
2.89
2.87
1.37
0.00
2x
DMSO-d_6, 500 MHz
2.00
2.00
6.00
2.00
2.00
18.00
10.0 9.0 8.0 7.0 6.0 5.0 4.0 3.0 2.0 1.0 0.0 -1.0
f1 (ppm)

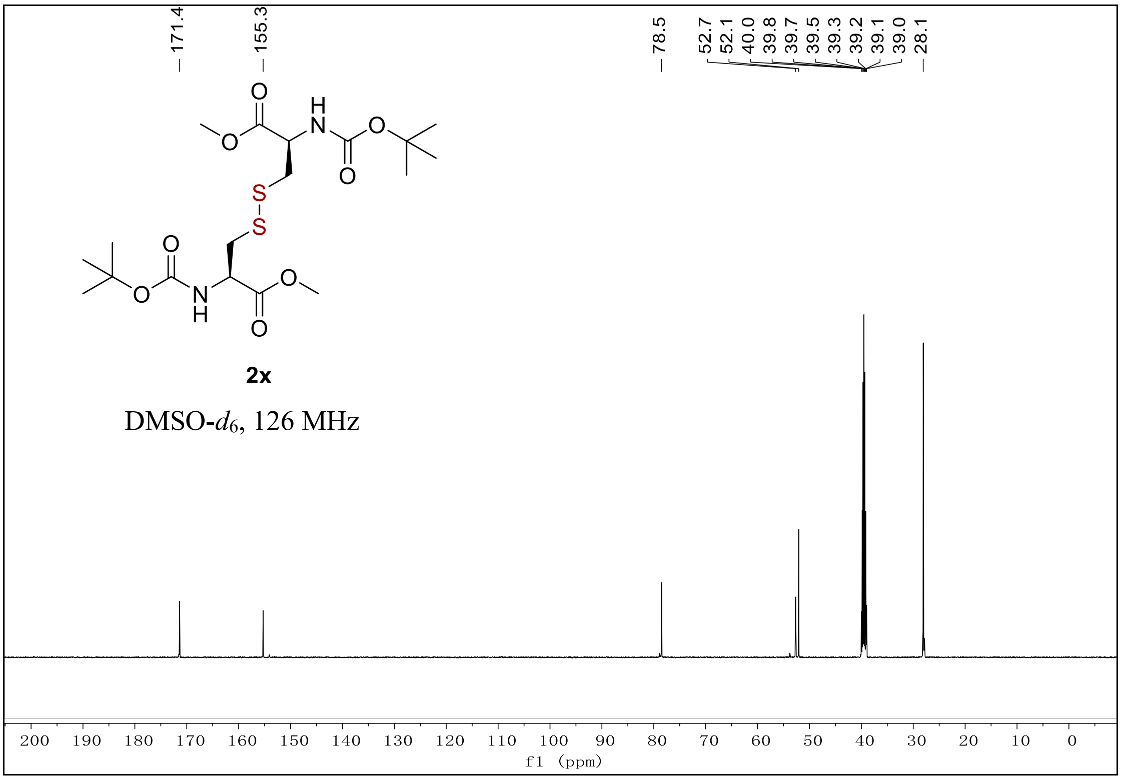
171.4
155.3
78.5
52.7
52.1
40.0
39.8
39.7
39.5
39.3
39.2
39.1
39.0
28.1
2x
DMSO-d_6, 126 MHz
200 190 180 170 160 150 140 130 120 110 100 90 80 70 60 50 40 30 20 10 0
f1 (ppm)

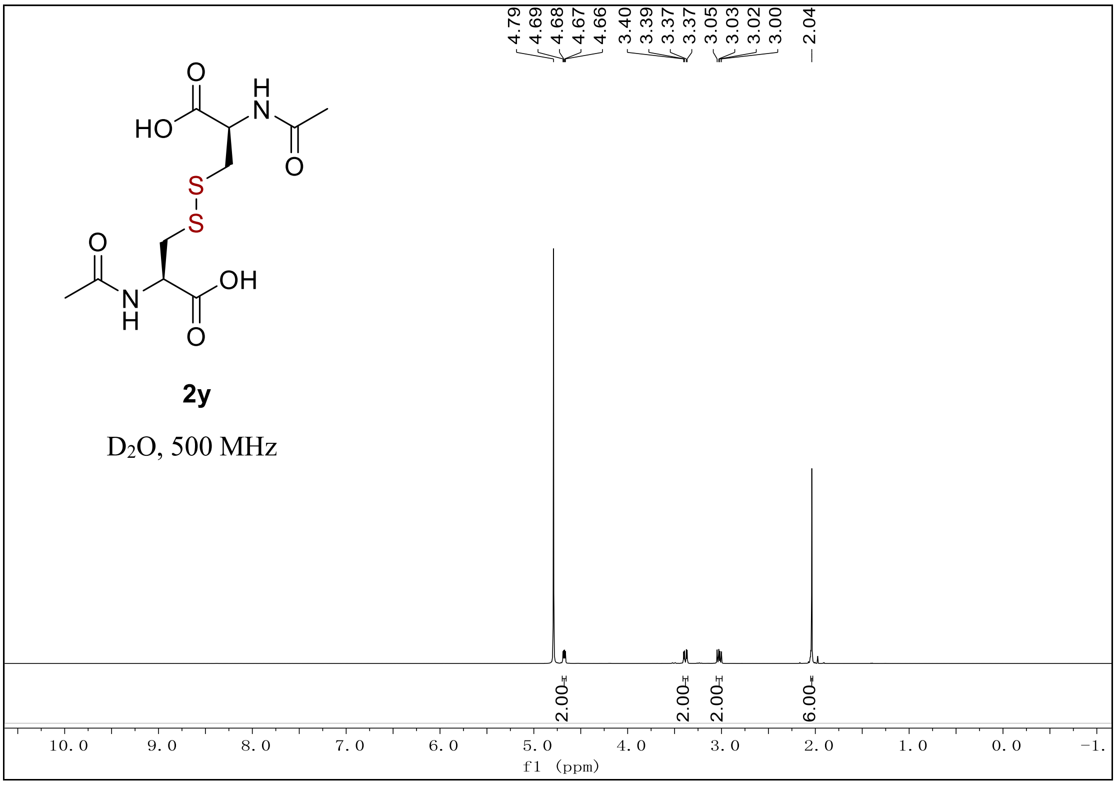
4.79
4.69
4.68
4.67
4.66
3.40
3.39
3.37
3.37
3.05
3.03
3.02
3.00
2.04
HO
OH
S
S
2y
D2O, 500 MHz
2.00
2.00
2.00
6.00
f1 (ppm)

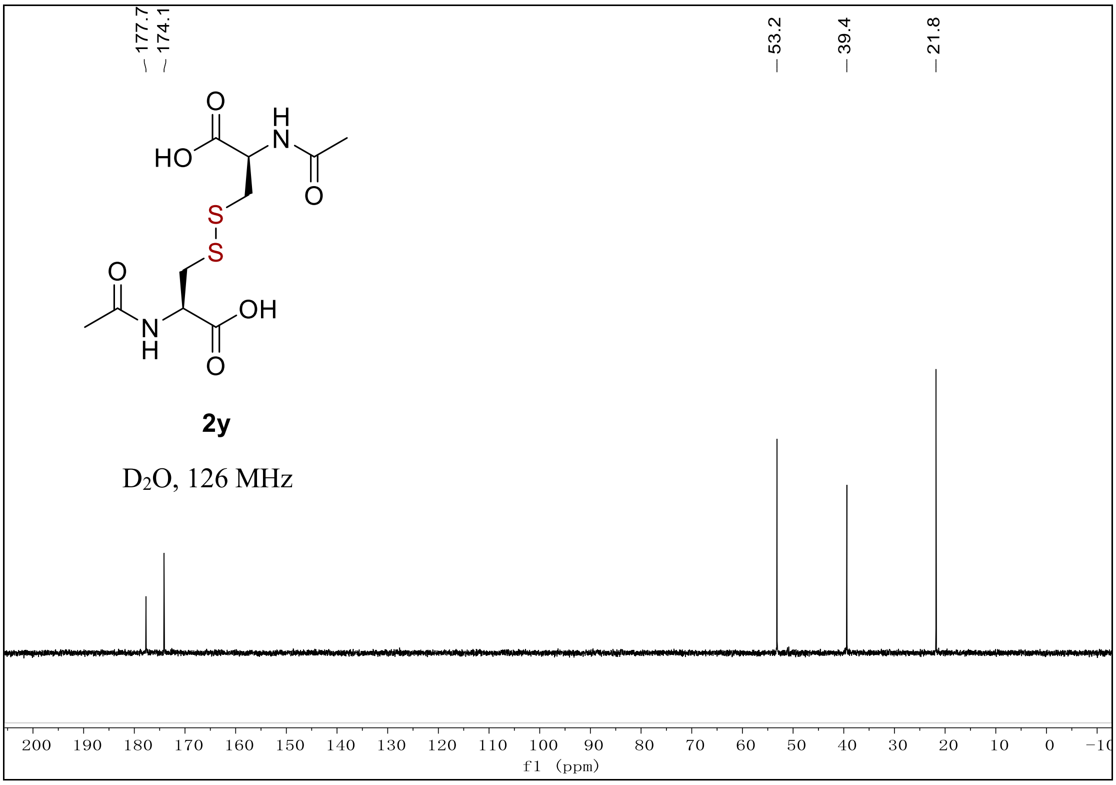
177.7
174.1
53.2
39.4
21.8
HO
OH
S
S
2y
D2O, 126 MHz
f1 (ppm)

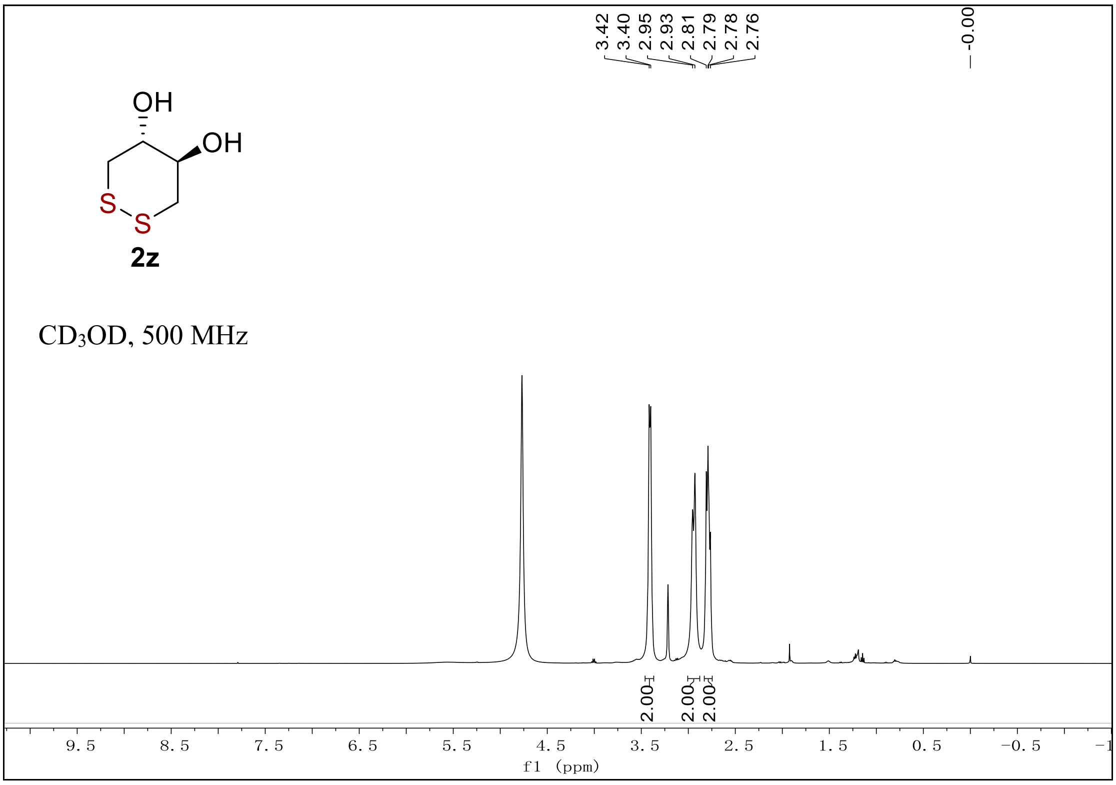
3.42
3.40
2.95
2.93
2.81
2.79
2.78
2.76
-0.00
OH
OH
S
S
2z
CD3OD, 500 MHz
2.00
2.00
2.00
9.5 8.5 7.5 6.5 5.5 4.5 3.5 2.5 1.5 0.5 -0.5
f1 (ppm)

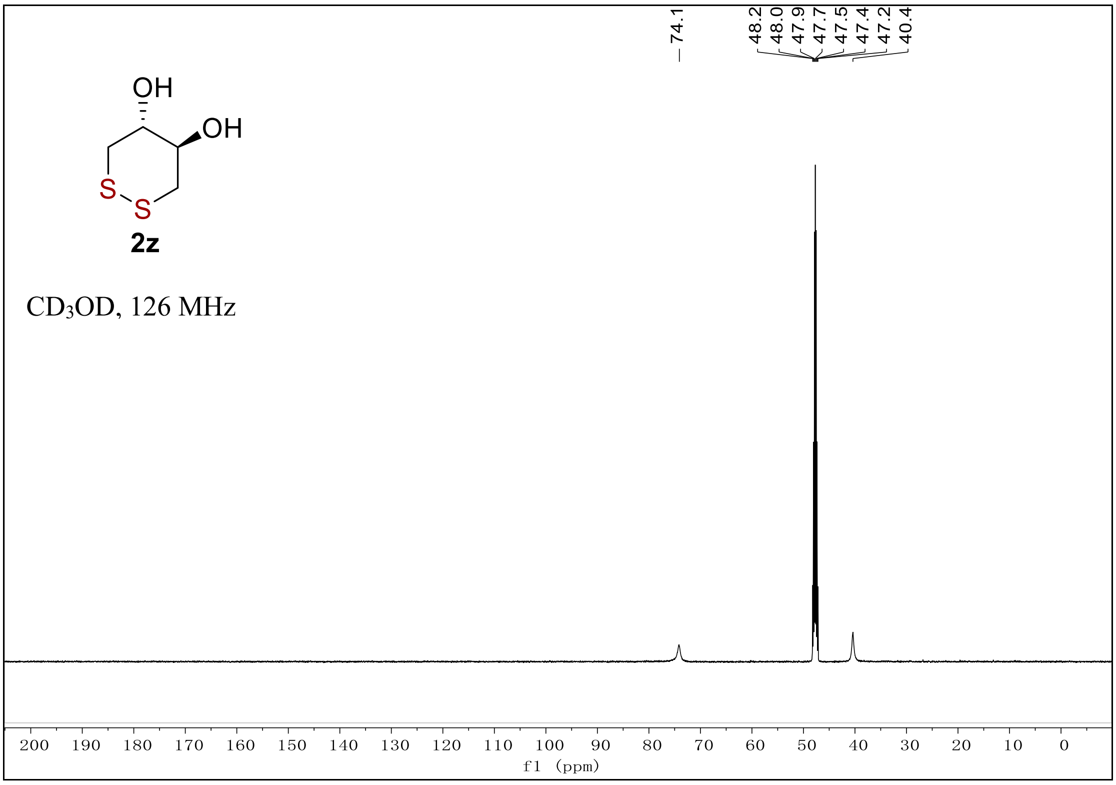
74.1
48.2
48.0
47.9
47.7
47.5
47.4
47.2
40.4
OH
OH
S
S
2z
CD3OD, 126 MHz
200 190 180 170 160 150 140 130 120 110 100 90 80 70 60 50 40 30 20 10 0
f1 (ppm)
